# Development of Life Course Exposure Estimates Using Geospatial Data and Residence History

**DOI:** 10.3390/ijerph22111629

**Published:** 2025-10-26

**Authors:** Stuart Batterman, Md Kamrul Islam, Stephen Goutman

**Affiliations:** 1 Environmental Health Sciences, University of Michigan, Ann Arbor, MI 48109, USA; kamrul@umich.edu; 2 Department of Neurology, University of Michigan, Ann Arbor, MI 48109, USA

**Keywords:** air pollution, epidemiology, exposure, geographical information system, geocoding, imputation, kriging, retrospective

## Abstract

Life course exposure estimates developed using geospatial datasets must address issues of individual mobility, missing and incorrect data, and incompatible scaling of the datasets. We propose methods to assess and resolve these issues by developing individual exposure histories for an adult cohort of patients with amyotrophic lateral sclerosis (ALS) and matched controls using residence history and PM_2.5_, black carbon, NO_2_, and traffic intensity estimates. The completeness of the residence histories was substantially improved by adding both date and age questions to the survey and by accounting for the preceding and following residence. Information for the past five residences fully captured a 20-year exposure window for 95% of the cohort. A novel spatial multiple imputation approach dealt with missing or incomplete address data and avoided biases associated with centroid approaches. These steps boosted the time history completion to 99% and the geocoding success to 92%. PM_2.5_ and NO_2_, but not black carbon, had moderately high agreement with observed data; however, the 1 km resolution of the pollution datasets did not capture fine scale spatial heterogeneity and compressed the range of exposures. This appears to be the first study to examine the mobility of an older cohort for long exposure windows and to utilize spatial imputation methods to estimate exposure. The recommended methods are broadly applicable and can improve the completeness, reliability, and accuracy of life course exposure estimates.

## 1. Introduction

### Importance and Applications of Geospatial Data 

Living near pollution sources has long been associated with high exposures to environmental contaminants, and proximity-based measures using residential and other locations have been widely used to explore associations between pollution sources and health effects [1,2,3,4]. In recent decades, many geospatial datasets have become available that contain information relevant to environmental exposures, including air quality (e.g., particulate matter, ozone), bioaerosols (e.g., pollen, virus), heat stress (temperature, humidity), land use and development (parks and “greenness,” industrial facilities, agricultural pesticide applications, highways), water and soil quality (radon, trace metals, harmful algal blooms), and noise. These datasets are derived using satellites, air and water quality measurements, administrative records, models, and other sources [5,6,7]. The broad coverage of geospatial data allows linking to residence or workplace locations from which individual-level and sometimes community-level exposure measures can be derived [4,5,8,9]. This presents many possibilities and advantages. In environmental epidemiology, this can boost the sample size and exposure contrasts, thus increasing the sensitivity to observe effects. It can add additional exposure types that increase the comprehensiveness of assessments and discover new relationships, and it can help account for local or small scale variation that reduces exposure measurement error [10]. Finally, geospatial data with a temporal dimension can be applied in spatial life course epidemiology [11], sometimes called spatio-temporal epidemiology [12], to approach the potential of the exposome and the quest to identify environmental factors affecting the onset and progression of disease. Health outcomes may be affected by the accumulation of exposure or the number of exposure events (deemed the accumulation of risk hypothesis); the timing of exposure, which is relevant to both susceptible developmental periods and the proximity to disease (the ‘recency’ hypotheses); and the changes in exposure over time due to exposure events, personal mobility, and other factors (the mobility hypothesis) [13]. Factors that lead to the accumulation of risk may be especially crucial for age-related diseases [14,15]. Understanding life course exposures and identifying culpable exposure sources can inform the development of health-protective interventions that reduce exposure, risk, and disease. 

The development of accurate life course exposure data is challenging. Despite rapid advances, biological monitoring providing ‘internal’ measures of exposure generally cannot provide temporal resolution or identify exposure sources, and many exposure types cannot be resolved [16,17]. Complementary ‘external’ exposure data using ground-based monitoring or satellite platforms prior to about 1980 to 2000 are very limited [14]. Even when available, ground-based environmental monitoring sites are spatially sparse, limited in spatial representativeness, and monitor relatively few parameters, leading to an inability to portray spatial patterns and provide residence- or individual-level information. Geospatial methods that integrate multiple types of observations and models can provide a viable method to quantify life course exposures, although developing accurate residence-based geospatial exposure estimates involves many choices and challenges [5].

The development of geospatial exposure estimates involves many steps, usually starting with residence histories that contain dates and addresses of study participants and sometimes workplace locations [10,18]. While long used to infer environmental exposures [3], residence histories typically are limited and incomplete [19,20]. Residential mobility, particularly moves between locations with different exposure levels, can increase exposure measurement error. Moreover, the likelihood of moving and the distance of moves can be affected by race/ethnicity, age, education, marriage status, work status, income, and other factors that may be associated with health outcomes, and thus can cause confounding [21]. In the second step, residence locations are geocoded to obtain spatial coordinates, ideally with positional accuracy [22,23,24]. A fundamental geocoding challenge here is missing, incomplete, erroneous, and incompatible data [19]; additional concerns include bias by geocoding method (e.g., parcel centroid, address point, or street segment), and clustering in missingness [25]. Third, a suitable geospatial dataset intended to represent a particular exposure type must be identified. This can involve choosing one or more pollutants (e.g., particulate matter under 2.5 µm diameter or PM_2.5_) or other stressors (e.g., heat, noise) available with an appropriate temporal and spatial coverage and resolution. Fourth, the exposure metric must be defined, which can include the concentration statistic (e.g., maximum, median, average), averaging time (e.g., daily, annual), and exposure window (e.g., 1 to 10 years before disease onset). A rarely considered temporal aspect is the exposure sequence, e.g., whether a PM_2.5_ episode preceded or followed an infectious disease outbreak. Fifth, the residential geocoordinates must be spatially linked to the location references associated with the geospatial data, e.g., point estimates, census tract or county level averages, rasters, or air pollution maps [22,26]. Most commonly, linking uses either spatial coincidence or unit-hazard analysis (e.g., the pollution level in the census tract hosting an individual’s residence) [27,28] or interpolations such as inverse distance weighting (IDW), radial basis functions (RBF), and kriging [29,30]. Sixth, the exposure metric or estimate is calculated, often as the residence time-weighted average concentration or the cumulative exposure experienced at residences in the exposure window. Lastly, the estimate may be refined by accounting for short- and long-time activity factors and behaviors (e.g., commuting patterns, time spent outdoors) [31], effects of microcompartments (e.g., attenuation of concentrations in buildings), and dosimetry (e.g., breathing rates). Methods to address each of these steps, and the data gaps and uncertainties therein, are needed to develop complete and accurate life course exposure histories.

This paper focuses on improving the reliability, accuracy, and completeness of life course exposure estimates based on geospatial data. We propose procedures for handling residence histories that contain missing and incomplete date and location information, including the use of a novel spatial imputation strategy. We also assess methods for linking geospatial data to air quality datasets and examine the variability and accuracy of the geospatial data. The techniques are applied to participants in a case/control study of amyotrophic lateral sclerosis (ALS), a disease typically affecting individuals in mid- to late-life. This older cohort is well suited for evaluating procedures relevant to life course studies.

## 2. Materials and Methods

### 2.1. Study Population 

Geospatial exposures were demonstrated using an ongoing epidemiological study that is investigating the risk and survival of ALS, a rare and devastating disease [32,33]. ALS patients were recruited during clinical visits to the University of Michigan Pranger ALS Clinic. The sole eligibility criterion was the ability to provide informed consent in English. Age- and sex-matched controls were enrolled across Michigan using postings on Facebook, the University of Michigan Health Research Platform, and random address mailings. Eligibility for controls included an English language requirement; exclusion criteria included a personal or family history of neurodegenerative disease in a first- or second-degree relative, an active infection, cancer, or autoimmune disease. Study participants completed a detailed questionnaire that included a residential history. Cases continued to receive clinical care at our center, thus information pertinent to ALS prognosis was abstracted from medical records, e.g., disease onset date and date of death. All participants provided written informed consent. The study was approved by the University of Michigan Institutional Review Board (HUM28826) and approved protocols were followed, including safeguards to protect participant privacy. 

### 2.2. Collection of Residential History Data 

Our goal was to obtain a full (birth to death or the present) residence history for each of the 1307 participants, who collectively indicated 9861 residential locations. In the survey, participants were asked the address of their current and all prior residences from birth, and the corresponding move-in and move-out months and years or, alternately, their age when move-in or move-out occurred. We also requested their birth date and relationship status. When information was incomplete or missing, our staff attempted to follow-up with the participant, family member, or caregiver. As expected, dates when a residence was occupied were often incomplete and sometimes inconsistent. As described below, we attempted to use all of the addresses provided by the participants to obtain a complete residential history.

We developed an approach to replace the missing year, month, and day data for both move-in and move-out dates. Considering the move-in year for a particular residence: (1) the move-in year was used if available; if not, then (2) the age at move-in plus the year of birth was used if this age was available; if not, then (3) the move-out year from the prior residence was used if available; and if not, then (4) the age at move-in for the prior residence plus the year of birth was used. For the first residence (typically the childhood residence), if the move-in date could not be determined, then the year of birth was assigned. Similar steps were taken for the move-out year, except that the timing was altered and for the latest (possibly the current) residence, the move-out year was set to the current year for living participants and the year of death for deceased participants. Months were handled similarly. Considering that most moves were completed in the summer, move-in and move-out months that could not be determined were set to August 1 and July 31, respectively. Since exposure estimates focus on long-term exposures, exact dates were not critical. 

A second pass of the date information identified gaps, overlaps, and other issues, after which mostly minor adjustments were made to improve completeness and consistency. First, we computed the gap or overlap period between consecutive move-in and move-out dates. If under two months or representing less than 20% of the total duration at these residences, the average was used to define the move-in and move-out dates. Second, the residential timeline of each participant was visualized (see supplemental information) to identify three special cases: (1) individuals with very short-term (< 1 year) and frequent (at least 3) consecutive moves, which mostly resulted from mobility following high school and college, e.g., potentially resulting from military service, and called ‘service homes’; (2) individuals reporting living in two residences simultaneously with one residence considered a ‘vacation’ home, defined by a rural or recreational-like location, e.g., upper Michigan or Florida, and the second in an urban location or city; and (3) individuals with large gaps in the record that were uncorrectable with available information. Third, the sequence of residences was checked and corrected as necessary; the visualization of the three cases above assisted this process. Finally, the completeness of the residential record was determined by summing gaps for each participant and expressing this sum as the percentage of their lifetime.

### 2.3. Geocoding and Spatial Imputation 

The geocoding analysis used a subset of participants and residential addresses, which included 4377 (97.3%) U.S. locations. (An additional 121 addresses were outside the USA and excluded from analysis.) This subset included addresses of participants occupied after the year 2000, reflecting the availability of the geospatial datasets described in the following section. To geocode, the address data from the survey was parsed, spell checked, and global substitutions were made for punctuation and abbreviations, e.g., “Ct” and “Ct.” were changed to “Court,” “S” was changed to “South,” and “Dr” was changed to “Drive.” Initial geocoding was performed using ArcGIS Pro 3.0.0. The addresses that did not geocode or that did have a 100% match (e.g., ties or unmatched addresses, see below) were manually investigated. Where possible, field errors were corrected, e.g., swapped cities and roads. Addresses listing cross streets were identified. All addresses were then reviewed for completeness, specifically for house/apartment number, street name, city, state, ZIP code, and country, and classified as follows: Category 1 was considered complete or sufficient for geocoding if the house number, road name, city, and state, or ZIP code were available, or if the provided cross streets were in residential areas. A missing state and ZIP code did not necessarily exclude an address if the individual’s prior or following address indicated the same or nearby city or state. Category 2 had insufficient data for geocoding due to missing essential information (e.g., house number, road name, or city) or used a non-standard address format (e.g., cross-streets given as the intersection of two roads, building or apartment building name). Most often, house/apartment numbers were missing. These addresses were imputed (described below). Category 3 had very limited and usually no information that allowed geocoding. 

Addresses in Category 1 (nominally sufficient for geocoding) were geocoded using ArcGIS Pro, 3.4.1 with the “Geocode Table” function and the “ArcGIS World Geocoding Service” as an input locator. The output summary indicated whether the address was matched, tied (a partial match that can correspond to multiple locations with the same or similar location name, or a result of spelling errors, omitted or incorrect information, or match with a large area such as a city), or unmatched (unable to find a corresponding address). The software allowed additional checks and updates to the input data to help confirm geocoding [26]. Tied and unmatched addresses required further handling and often additional information for precise geocoding, and were placed in Categories 2A and 2B, respectively, for manual checking using Google Maps and Bing Maps, which frequently identified incorrect or omitted information (e.g., ZIP code, city, and part of the street name, such as “Terrace” or “South”, or an address for an apartment complex). Using the revised address, geocoding results from ArcGIS Pro, Google Maps, and Bing Maps were compared, and if two of the three results agreed (<15 m discrepancy for complete addresses and within the parcel boundary for buildings and building complexes), geocoding was considered complete. 

The remaining addresses in Category 2 were further classified. Category 2C addresses were specified as cross-streets or apartment complexes that allowed geocoding to 1 km resolution or better. These were geocoded and verified as described for Group 1. Category 2D addresses could not be accurately geocoded due to missing information, e.g., house number. Category 2E addresses had only a name for an apartment complex, area, military base, or small town. 

An imputation strategy was developed for Category 2D and 2E addresses following the philosophy of multiple imputation (MI) procedures that yields unbiased estimates by generating an ensemble of estimates for each missing datum that has the same distribution as the known data [34]. In the spatial context, this recognized that a single imputation was unlikely to reflect the average due to local spatial variation, e.g., a road segment midpoint or a city centroid would not reflect locations near a busy highway or an industrial area. The imputation procedure made three assumptions. First, the true location of the residence was equally likely in any residential area along the smallest road segment or area that could be delineated by the available information. Second, given the correlation expected between exposures at nearby sites, locations separated by not more than the resolution of the geospatial dataset were sufficient, e.g., the air pollution datasets described below have ~1 km resolution, thus imputation locations spaced closer than 1 km provide little gain. Third, only addresses that could be constrained to a road segment under 20 km in length or an area under 20 km^2^ were imputed to avoid the possibility of large errors. These assumptions can be adjusted to match specific datasets.

**Figure 1:**
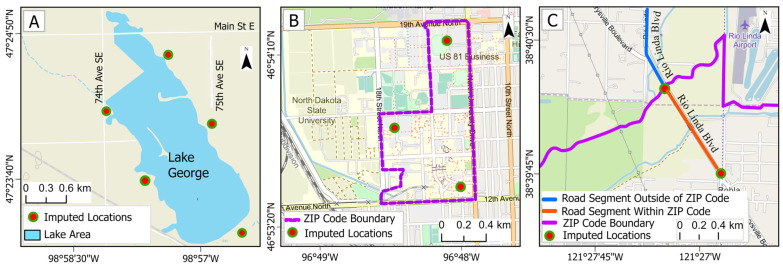
Examples of three imputation strategies: (**A**) Address given as a housing, community, or lake name; (**B**) address given as a small town constrained by the ZIP code; and (**C**) address given as a road constrained by the ZIP code. Orange circles show the imputed locations. The locations are hypothetical.

Figure 1 depicts the imputation procedure for three cases. For addresses containing road names but not house numbers, the road length was determined using Google Maps. Constraints imposed by city or ZIP code boundaries were considered, and uninhabited portions (e.g., industrial areas, water bodies) along the road segment were excluded. Segment endpoints were determined and used to determine the segment’s length (for straight roads). Next, intermediate geocoordinates were placed every ~1 km along the segment in residential areas, e.g., a 3 km long segment had four geocoordinates. For short road segments (< 1 km in length), the centroid was selected. For curved or circular roads, geocoordinates were selected in residential areas with a separation distance of ~1 km. For addresses missing road names or house numbers, but where a town, apartment complex, or other area was specified, the residential area was calculated, again using any constraints imposed by the administrative or ZIP code limits. The number of locations selected for areas depended on the inhabited area (water and uninhabited areas were excluded), e.g., one (centroid) was used for areas < 1 km^2^, and three or four geocoordinates for 2–5 km^2^ areas. Next, the pollutant level at each geocoordinate was estimated (described later). The final estimate used the levels at the multiple geocoordinates or the average of these values. We calculated the variability of the MI estimates at each (missing) location as the coefficient of variation (COV) and reported the mean and 95th percentage confidence interval (95% CI) across the 50 sites and the 17–19 years available for each pollutant.

The imputation procedure was evaluated for the five exposure types described below. For each, 50 randomly selected and complete addresses were selected and house numbers removed. The MI procedure used a total of 263 imputation locations for these addresses (~5.1 locations per address). Imputed estimates were compared to “true” values at the known geocoordinates using scatterplots, distribution checks, and Shapiro–Wilk, Mann–Whitney U, and Kolmogorov–Smirnov (KS) tests.

### 2.4. Exposure Datasets 

**Figure 2:**
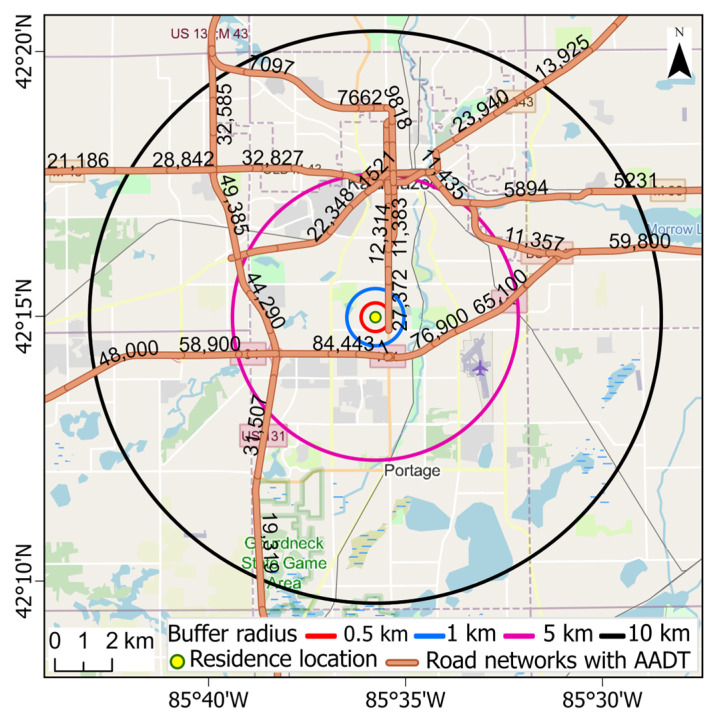
Example of road network with AADT and circular buffers of 0.5, 1, 5, and 10 km radii for calculating traffic intensity information around a hypothetical residence location. Numbers show AADT on road segments.

Five measures of air quality and traffic-related air pollutants were considered: PM_2.5_, black carbon (BC), nitrogen dioxide (NO_2_), and non-commercial and commercial traffic intensity (TI). The PM_2.5_ and BC datasets were derived from satellite data and chemical transport modeling using GEOS-Chem calibrated to ground-based observations with a resolution of 0.01 degrees (~1 km) [35]. The netCDF file format was downloaded (https://sites.wustl.edu/acag/surface-pm2-5-archive; accessed on 12 January 2025) and converted to point-based data. The NO_2_ data was obtained from the Center for Air, Climate and Energy Solutions [36], which provides annual average concentrations from 1979 to 2020 at the census block-group level derived from land use regression (LUR) complemented with satellite data for later years [37]. The TIs for light and heavy duty vehicles (LDVs, HDVs), surrogates of traffic-related air pollutants [38,39,40,41], provided “hyperlocal” information for the Michigan sites. The TIs were derived by first defining circular buffers of 0.5, 1, 2.5, 5, and 10 km radii around each residence location (Figure 2). Second, the total road length (km), associated annual average daily traffic (AADT) and annual average commercial daily traffic (CAADT) in the buffer were extracted from shapefiles and data obtained from the Michigan Department of Transportation (https://gis-mdot.opendata.arcgis.com; accessed on 21 January 2025). Third, LDV vehicle kilometers traveled (VKT) in each buffer was estimated by subtracting CAADT from AADT for each segment, multiplying this value by the segment’s length, and then summing these products across segments in the buffer. Fourth, VKT in disjoint concentric rings was obtained by differencing VKTs for the next smaller buffer, and the TI was calculated as the sum of VKTs for the 0.5, 1, 5, and 10 km rings weighted by 1.0, 0.5, 0.1, and 0.05, respectively, reflecting the attenuation of pollutants at longer distances. These weights are for illustrative purposes, and more specific weights can be developed using dispersion models and site-specific meteorological data. Lastly, the TIs were calculated at the locations of surface level monitoring sites (described below) and for a 1 km grid in Michigan. The TI for commercial vehicles (CVKT) was calculated similarly using CAADT. Most of comparisons of these datasets used a single year to avoid the issue of temporal trends, and the year 2016 was selected as it had a high number of BC monitoring sites (48 sites with sufficient data for annual averages).

### 2.5. Spatial Linking, Interpolation, and Correlation

The agreement between the air pollution datasets and annual average measurements of PM_2.5_, BC and NO2 at monitoring sites across the US collected by the US Environmental Protection Agency (https://aqs.epa.gov/aqsweb/airdata/download_files.html; accessed on 21 January 2025) was characterized using several performance measures, e.g., root mean square error (RMSE), mean average error (MAE), mean absolute percentage error (MAPE), coefficient of determination (R^2^), and scatterplots. Only monitoring sites with at least 75% completeness were considered, which reduced the number of PM_2.5_, BC, and NO_2_ sites from 945, 65, and 459, respectively, to 492, 48, and 409. This analysis required converting the PM_2.5_ and BC datasets to point data using the “raster to point” function in ArcGIS Pro. (The NO_2_ dataset was already in point format.) Results for 2016 are presented, although other years were tested. We compared observed and estimated data using log probability plots, correlations, and Bland–Altman plots.

The evaluation above also required spatial linking to match locations of the monitoring sites, which provided an opportunity to evaluate various interpolation techniques. For consistency, the evaluation used kriging as the interpolation method. However, we also tested IDW schemes with the distance raised to powers from 1.0 to 1.8 using the “geostatistical analyst tools” in ArcGIS Pro, RBF interpolations (including completely regularized spline, inverse multiquadric, and multiquadric kernel functions), and ordinary and universal kriging (Gaussian, exponential, spherical kernel functions) using the “geostatistical wizard” in ArcGIS Pro. Each interpolation type was tested using from four to sixteen points. 

To further evaluate the spatial resolution needed to evaluate roadway increments and other local influences on pollutant levels, PM_2.5_, BC, and NO_2_ concentrations were plotted against the distance between each monitoring site and the nearest major road determined using the “near” function in ArcGIS Pro and the National Highway Planning Network shapefile from the U.S. Department of Transportation (https://data-usdot.opendata.arcgis.com/datasets/usdot::national-highway-planning-network/about; accessed on 6 January 2025). 

The spatial variability of the exposure metrics was evaluated at several scales. At the large or regional scale, concentration distributions for observed (monitored) and estimated (geospatial) data were compared. At the local scale, the coefficient of variation (COV) of the geospatial data at the pixel level within 1 and 2.5 km of each monitoring site was calculated. Additionally, in the kriging program, we generated covariograms, range and sill statistics, and constructed correlation maps for two spatial domains (southeast Michigan, Michigan) and for various correlation functions (spherical, Gaussian, exponential, circular, tetraspherical, pentaspherical, rational quadratic, stable). The RMSE of the kriging estimates was calculated using eight to sixteen neighbors, 100 lags, and a lag distance of 1.5 km. Most of these analyses used the “geostatistical wizard” tool in ArcGIS Pro.

## 3. Results

The average age of participants at study enrollment (consenting) was 63.0 years (N = 1307; range: 20.3 to 92.7 years; Supplemental Table S1). The cases lived only 2.16 ± 1.82 years beyond consenting (N = 512). By design, the ages of cases and controls were matched. The cases and controls showed several differences: cases were more likely to be married or with a partner than controls (79 versus 66%), more likely to be widowed (6.7 versus 4.7%), and less likely to be divorced or separated (9.6 versus 17.5%). Males represented 54.9% of the cohort. Compared to females, males were more likely to be married or with a partner (80% versus 66%), and less likely to be widowed (2.2% versus 9.7%), divorced or separated (10.6 versus 16.1%), and to have been never married (6.1 and 7.5%, respectively). These differences were unlikely to affect our assessment of exposures and the protocols described below, thus the following assessment pooled cases and controls. However, residential mobility did differ by age and sex, thus these factors were examined. 

### 3.1. Residential Dates

Complete information (month and year) was provided for 76.8% of move-ins and 65.6% of move-outs; years (only) were provided for 88.5 and 76.7% of move-ins and move-outs, respectively. Participants provided their age for 15.3% of move-ins and 15.4% of move-outs. Overall, 28.8% of the month and year information required to construct a residential history was missing.

The frequencies of strategies used to impute dates are shown in Table 1. For example, of the 9861 move-in addresses, the year of move-in was provided in 8731 cases; if unavailable, the move-in age plus the date of birth was used (N = 806); and if these were unavailable, the prior move-out year was used (16 cases). The second pass of the data identified 1160 cases (11.8% of the total) when gaps or overlaps between adjacent residences exceeded 2 months. Many of these were corrected in the second pass, leaving 634 cases (6.4%) with gaps or overlaps that exceeded our criteria (2 months or 20% of the duration of prior and following residence periods). These cases were investigated individually, which identified vacation homes (N = 50), typically occupied by older adults (roughly 50–60 years of age), and a smaller number of individuals occupying a series of short-duration or service homes (N = 9), most commonly by young adults (20–35 years). The year of move-in or move-out could not be established in 267 cases. Overall, the approach successfully completed 98.7% of the dates for residential moves. The following used the imputed dates. 

### 3.2. Residence Locations

**Figure 3:**
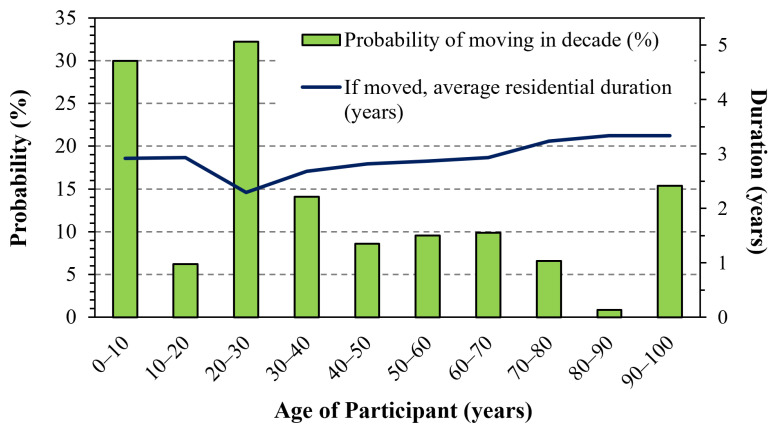
Probability of moving by participant age, and average residential duration if moved. N = 1307 (total), but sample size declines with age and N = 963, 503, 116, and 13 for the last four decades.

The residence locations were mostly in Michigan (78%, 3123 locations) but many (22%, 889 locations) were elsewhere in the US (Supplemental Figure S1 displays locations.) The participants reported an average of 7.55 residence locations per person (range: 1 to 18). The number of residences per person differed slightly by sex and marital status (Table 2), e.g., females who never married had the highest number of residences (lifetime average of 8.20 per person). For the 20 years prior to consenting, participants lived in an average of 2.16 homes, and individuals who were never married, divorced, or separated lived in approximately one additional residence compared to those married or living with a partner. Figure S2 gives an example of the visualizations of the residence time history for a fairly mobile participant (12 addresses); such displays were helpful in verifying dates and moves. Trends in the number of residences per person by age showed small differences (Figure S3) except for the youngest (< 35 years of age), who reported a median of only 5.2 residences per person. Differences by sex were small except for individuals aged 65 to 70 years where females reported one or two more residences per person than males. The likelihood of moving averaged 11.6% per person per year and depended strongly on age (Figure 3). Moves were most likely when participants were very young (30% per person per year when 0 to 10 years old) and as young adults (32% when 20 to 30 years old). These trends suggest that, generally, people in this older cohort had completed most of their residential moves earlier in life. 

**Figure 4:**
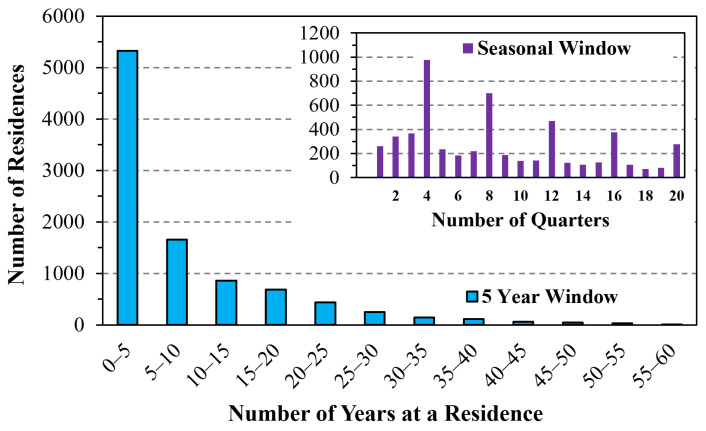
Distribution of time spent at a residence. Inset plot shows results for times below 5 years by quarter of year.

The mean duration at a residence averaged 8.15 ± 9.69 years and varied greatly (Figure 4). Slightly over half of durations were under five years (increments of 1, 2, 3, 4, or 5 years represented 20.2, 13.8, 9.7, 7.6, and 5.6% of moves, respectively); durations exceeding 10 years represented 27.6% of moves. The duration of the last (or current) residence was long, averaging 20.5 ± 14.0 years. 

**Figure 5:**
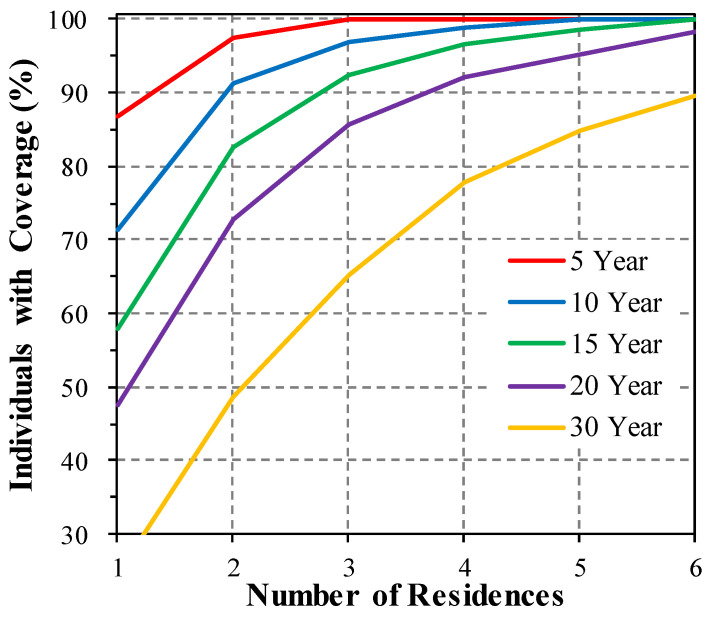
Fraction of individuals with complete residence histories for exposure windows from 5 to 30 years by number of most recent residences. Windows are defined as years prior to consenting.

The number of residences needed to capture exposure windows from 5 to 30 years in length is shown in Figure 5. The past 20 years, for example, required information for the past five residences to complete the residential history for 95% of participants; a 10-year window required three residences. (The other 5% of participants had additional moves.) This information can help design survey instruments that are efficient by reducing participant burden, i.e., asking about the last five residence locations might be sufficient for most applications. However, obtaining information for all locations within the exposure window is recommended to avoid omissions and possible bias. 

### 3.3. Geocoding 

Of the 4377 addresses considered in the geocoding subset, 2948 (67.4%) had sufficient information for precise geocoding with results confirmed by at least two geocoding systems (Category 1); an additional 49 locations (1.1%) could be geocoded after minor address corrections (Category 2A; Table 3). Locations could be localized to within 1 km for an additional 383 (8.8%) of residences by using or correcting available information (Categories 2C and 2B, respectively); these were mostly apartment buildings or complexes, dormitories, and short roads. The spatial imputation procedure was applied to the remaining 632 records (14.4%), which included road segments less than 20 km in length (407 residences, 9.3%, Category 2D) and areas less than 20 km^2^ in area (225 residences, 5.1%, Category 2E). An average of 5.9 locations was used to impute each residence location (total of 3769 geocoordinates). Lastly, 365 addresses (8.3%) could not be geocoded with available information (Category 3), mostly due to missing or inconsistent street addresses or other essential information (245 residences). Category 3 residences were often childhood homes, suggesting recall issues (also seen for early life move-in/out dates). These residences were widely dispersed (occurring in 44 states), and males and females had similar likelihoods (3.8 and 4.3%, respectively), suggesting non-differential bias. Overall, information provided by participants allowed 68.5% of addresses to be precisely geocoded, 8.8% to be localized to 1 km and geocoded, and 14.4% to be imputed, boosting the overall geocoding completion rate to 91.7%. 

### 3.4. Spatial Imputation 

**Figure 6:**
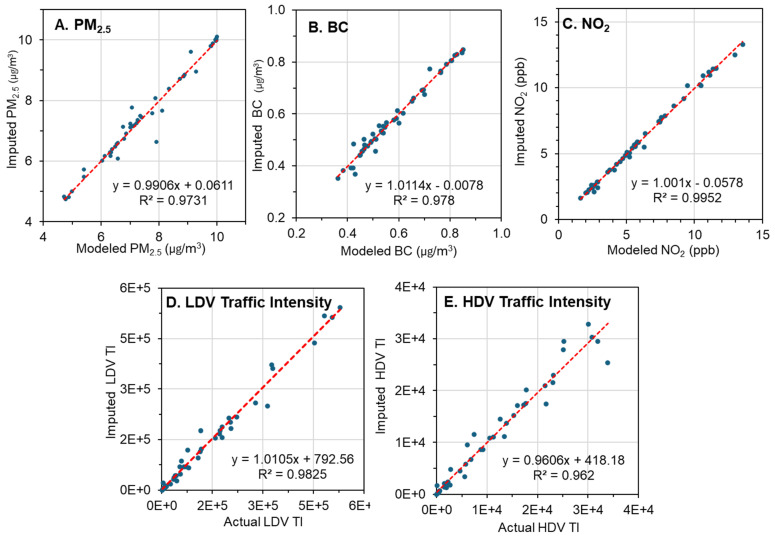
Scatterplots comparing imputed data to actual values, linear trend, and R_2_ for PM_2.5_, BC, NO_2_, and Traffic Intensity (TI). N = 50 for each figure. Regression line and R^2^ indicated.

Because PM_2.5_, BC, NO_2_, and TI levels in the test datasets were not normally distributed, non-parametric tests were used to evaluate the agreement between known and imputed values. Scatterplots for the five measures showed good agreement (Figure 6), high Spearman correlation coefficients (0.92 to 0.99), and Mann–Whitney and KS tests showed no statistically significant differences (Table 4) as did distribution plots (Figure S4). The variability of the MI estimates varied by pollutant and location, e.g., for PM_2.5_, the COV across sites and years averaged 1.8%; (95% CI: 0.0–8.6%); NO_2_ averaged 6.8% (95% CI: 0.3–26.4%), and BC averaged 2.7% (95% CI: 0.1–11.3%). These results suggest that the spatial imputation procedure reflected the uncertainty at the test locations and provided representative estimates for the exposure metrics. 

### 3.5. Comparison of the Geospatial Datasets

**Figure 7:**
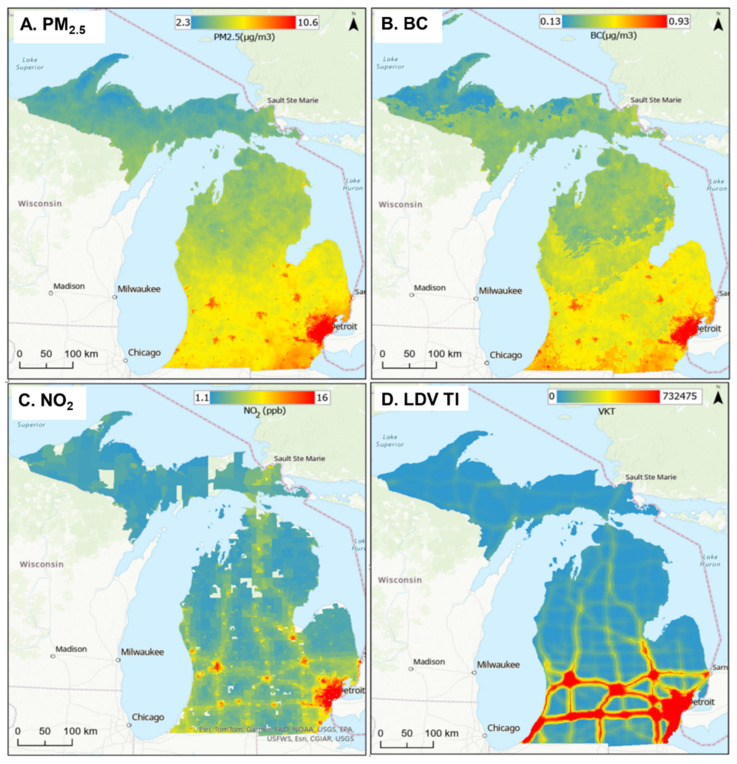
Maps showing levels of (**A**) PM_2.5,_ (**B**) BC, (**C**) NO_2_, and (**D**) non-commercial traffic intensity (TI) across Michigan for 2016. Concentrations are in µg/m^3^. TI uses inverse-distance-weighted VKT in a 10 km buffer.

**Figure 8:**
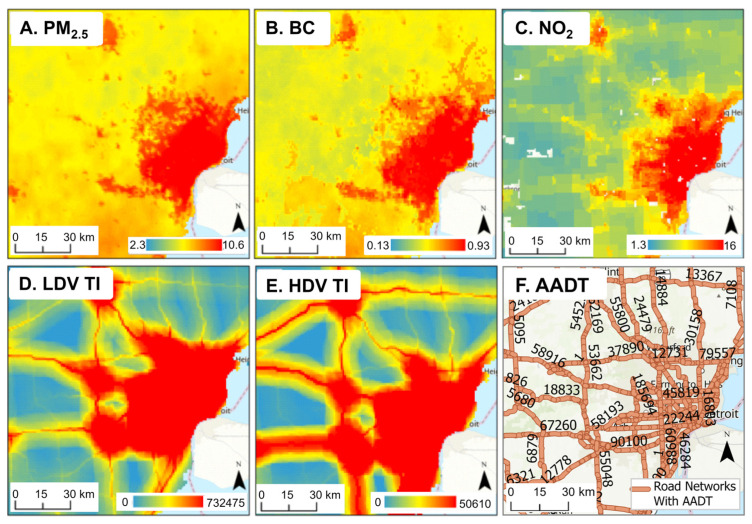
Maps showing levels of (**A**) PM_2.5,_ (**B**) BC, (**C**) NO_2_, (**D**) LDV and (**E**) HDV traffic intensity (TI), and (**F**) road network with AADT (vehicles per day) in the Detroit metropolitan area for 2016.

PM_2.5_, BC, NO_2_, and LDV TI levels are mapped across Michigan in Figure 7 and at greater detail for urban southeast Michigan in Figure 8. (Figure S5 provides the map for HDV TI.) The levels are correlated with population density, industrial activity, and traffic, highest in the Detroit area (containing over 4.3 million people), and lowest in the sparsely populated Upper Peninsula. Given their relatively long atmospheric lifetime, PM_2.5_ and BC had modest spatial gradients, while NO_2_ and especially TI levels had sharper gradients. The five exposure types were moderately to highly correlated across the state (R = 0.58 to 0.96; Table 5), but correlations in southeast Michigan area were lower, especially between PM_2.5_ and BC with TI (R = 0.23 to 0.40), reflecting additional PM_2.5_ and BC emissions from industry and other pollution sources not reflected in TI, a smaller urban–rural gradient, and effects of fine-scale gradients. As expected, HDV and LDV TI measures were highly correlated at the two scales (R = 0.92 to 0.96). The high correlation between NO_2_ and TI at the urban scale (R = 0.77 to 0.78) reinforces the primacy of vehicle traffic as an NO_2_ source. Overall, this analysis shows a high correlation between PM_2.5_ and BC and between LDV and HDV TIs at both state and urban scales, somewhat different spatial patterns between the three pollutant groups (PM_2.5_/BC, NO_2_, and LDV/HDV TIs), and effects of spatial scale.

### 3.6. Comparison of Geospatial and Monitoring Data 

**Figure 9:**
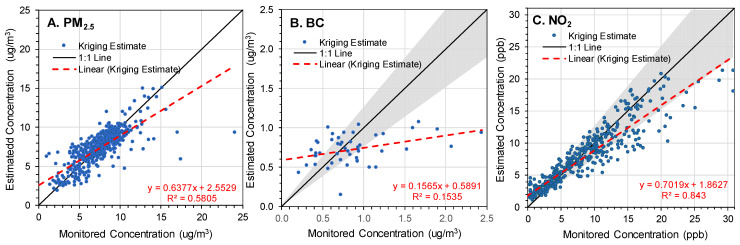
Scatterplots of geospatial estimates (interpolated using kriging) versus monitored data for (**A**) PM_2.5_, (**B**) BC, and (**C**) NO_2_. Annual averages for 2016. Gray area shows region within ±25% of the 1:1 line. Regression line and R^2^ shown in red. N = 492, 48, and 409 for PM_2.5_, BC, and NO_2_, respectively.

**Figure 10:**
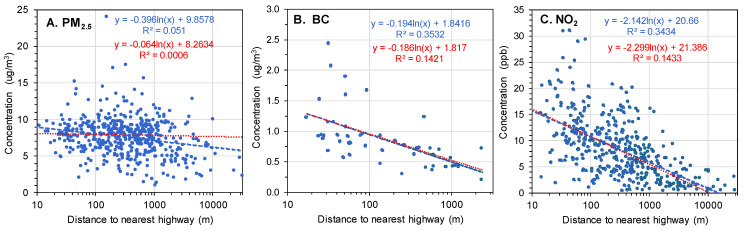
Scatterplots of monitored concentrations versus distance to nearest highway for (**A**) PM_2.5_, (**B**) BC, and (**C**) NO_2_. Annual averages for 2016. Regression line and R^2^ shown for full dataset (blue line, blue text) and for distances less than 500 m (red line, red text). Most BC monitors were near roads or in urban areas, and none were more than 3000 m from the nearest highway.

The agreement between annual average measurements of PM_2.5_, BC, and NO_2_ at monitoring sites and the geostatistical data extracted for the same locations (Figure 9) was moderately high for NO_2_ (R^2^ = 0.84, slope = 0.70, 64% of sites within ±25%) and PM_2.5_ (R^2^ = 0.58, slope = 0.64, 83% of sites within ±25%), but low for BC (R^2^ ≈ 0.15, slope = 0.15, 39% of sites within ±25%). The higher concentrations of NO_2_ and PM_2.5_ were systematically underpredicted. Other years showed comparable results for PM_2.5_ and NO_2_, while results for BC were more variable (Figure S6). The performance for NO_2_ and PM_2.5_ (but not BC) was similar to that discussed by the dataset developers [35,37]. The discrepancies for BC may be attributed to the small sample size (N = 48), selective placement of the BC monitors near roads, outliers in the observed data, and most importantly, spatial averaging across the pixel area (1 km^2^) that did not resolve small scale features, specifically elevated levels near major roads, i.e., the “roadway increment.” Figure 10B,C shows this increment for BC and NO_2_ and show the strongest effects within ~100 m of the road. Trends were similar in other years and, again, BC showed more variability (Figure S7). Roadway increments were muted for PM_2.5_ (Figure 10A). The monitoring dataset included many BC monitors placed near highways (29% were within 50 m), compared to PM_2.5_ and NO_2_ monitors (11 and 12%, respectively; Table S2). (Monitors in the EPA near-road network were placed within 50 m of large roads.) Levels of traffic-related air pollutants near major roads were elevated to only a distance of ~1 km or less from the road. Because the geospatial estimates did not capture spatial variation below a resolution of 1 km, the agreement with monitored data was poor for BC, particularly given the large fraction of near-road BC monitors. Similarly, the NO_2_ roadway increment was not accurately portrayed, but the agreement between geospatial and monitored levels (Figure 9C) did reflect regional scale (e.g., urban-nonurban) differences, as well as the few NO_2_ monitors near major roads. 

**Figure 11:**
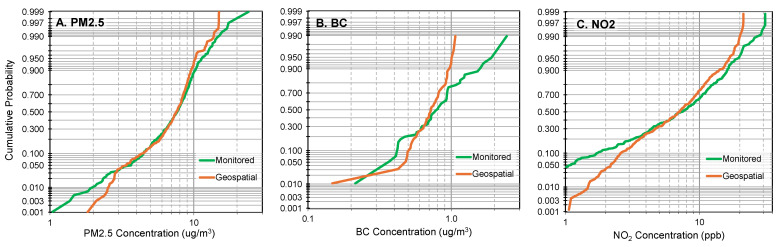
Log probability plots for (**A**) PM_2.5_, (**B**) BC, and (**C**) NO_2_ contrasting monitored annual average concentrations to geospatial estimates.

Figure 11 shows distributions of monitoring data and the corresponding geospatial estimates. These probability plots in this regional scale (US-wide) analysis reveal that the geospatial data was compressed, specifically, the top quintile of geospatial estimates was biased downwards for the three pollutants while the bottom quintile was slightly biased upwards for PM_2.5_ and NO_2_. The Bland–Altman plots for the same comparison, an analysis that matches by location (Figure S8), revealed that all high concentration values of BC were consistently underpredicted, NO_2_ had a similar trend but considerable variability (e.g., underpredictions at high concentrations are larger and more common than overpredictions), while the PM_2.5_ trends were driven by a few outliers. These biases reduced the spatial variability of the geospatial data, e.g., COVs of the geospatial data were only 29, 25, and 36% for PM_2.5_, BC, and NO_2_, respectively, compared to 34, 53, and 69% for the monitored data (Table S3). This compression or shrinkage mainly affected the tails of the distribution, e.g., the highest and lowest 1 to 10% of the data, and likely the reason that KS tests did not show differences in the distributions of the geospatial and monitored data for PM_2.5_ (P = 0.11, N = 492) and BC (P = 0.16, N = 48), although NO_2_ differed (P = 0.05, N = 409). The key significance of this compression is that the geospatial data may not identify the polluted sites.

Spatial variation at the local scale, e.g., within a few km, cannot be characterized using the ambient monitoring observations given the spatial sparseness of sites. The analyses of geospatial data within a 2.5 km radius around each monitoring site (16 pixels) yielded COVs averaging 4.8, 6.1, and 8.3% for PM_2.5_, BC, and NO_2_, respectively, and slightly lower (3.0, 4.3, and 6.2%, respectively) for a 1 km radius (4 pixels; Table S3). The variation depended on site, e.g., COV distributions were skewed rightwards, COVs exceeded 20% at a few sites (Figure S9), and some dependence on concentration was noted (Figure S10), e.g., COVs for BC tended to decrease at higher concentrations. Overall, the geospatial data varied smoothly and gradually in space; local variation was only 10 to 20% of that seen at the regional level and, as seen earlier (Figure 10), small-scale variation from local sources like major roads was not portrayed.

**Figure 12:**
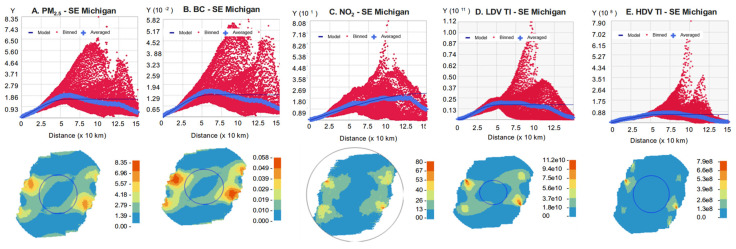
Semivariograms for (**A**) PM_2.5_, (**B**) BC, (**C**) NO_2_, (**D**) LDV TI, and (**E**) HDV TI using best-fit correlation model and southeast Michigan domain. Y is ½ covariance. Binned values (red dots) use square cells that are one lag wide. Average values (blue crosses) are generated by binning points within angular sectors. Model line fits binned values. Map shows angular and range as circles. Uses 2016 geospatial data.

**Figure 13:**
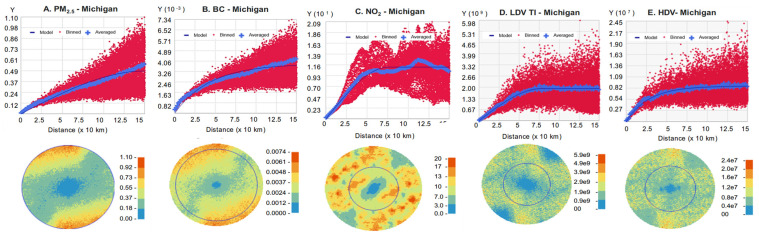
Semivariograms for (**A**) PM_2.5_, (**B**) BC, (**C**) NO_2_, (**D**) LDV TI, and (**E**) HDV TI for all of Michigan. Otherwise, as Figure 12.

Semivariograms for the five metrics in southeast Michigan and throughout Michigan are shown in Figure 12 and Figure 13, respectively, and kriging results are summarized in Table 6. The results depended on the dataset and fitting parameters, including the correlation function and the spatial domain. The estimated range (distance beyond which correlation is negligible) was typically 50–150 km. Interestingly, covariograms and ranges for the TI measures resembled those for the pollutants, although the TI measures were based on only a 10 km radius; the correlation at longer distances reflected the layout of the road network. All semivariograms showed negligible variation for separation distances below a few km. The variogram maps also suggested directional effects, e.g., sharper gradients in the north–south direction at the state level, reflecting the gradients across the region (Figure 7). The geospatial datasets contained no information below 1 km separation distance. These results suggest that the geospatial datasets primarily reflect variation between urban and nonurban environments.

In summary, the geospatial datasets represented the regional trends of observed PM_2.5_ and NO_2_ levels, although the variability of both upper and lower concentrations was attenuated. At the local scale, the geospatial data had little variability and did not reflect impacts from local sources, including the near-road increments seen for NO_2_ and especially for BC. 

### 3.7. Interpolation and Linking

The performance of the interpolation schemes used to estimate pollutant levels at the monitoring sites from the geospatial data is summarized in Table S4. Kriging and the inverse multiquadric RBF had marginally better performance for BC, and kriging and the multiquadric RBF had slightly better performance for NO_2_. However, the differences were small, e.g., nearest neighbor and kriging estimates were nearly the same. This insensitivity to the interpolation scheme primarily resulted from the limited local-scale variation in the geospatial datasets, as discussed above, and the short interpolation distances (< 1.5 km) in this application.

## 4. Discussion

This study has developed and evaluated several methods to improve the completeness, accuracy, and interpretation of residence-based life course exposure estimates derived from geospatial datasets and residence histories. The methods focused on resolving gaps and errors in dates, imputing residence locations from incomplete location data provided by study participants, linking these locations to geospatial datasets, selecting datasets, and interpreting estimated exposure data. These concerns are important given the growing use of geospatial data in environmental epidemiology, including those studies examining large populations and especially those examining vulnerable and disproportionately exposed populations, e.g., low-income and minority persons living near industry and major roads. They are also important for studies of rare diseases, e.g., amyotrophic lateral sclerosis (ALS) [32,33] where geospatial applications are just emerging [42,43,44]. The association of ALS risk and survival with exposure to air pollutants and other exposures across the life course highlights the need for improved localization and quantification of environmental hazards and exposures, benefiting both retrospective and prospective studies. Furthermore, geospatial analyses might help identify clusters of disease cases and determine if they are linked to specific emission sources or activities [45]. While beyond the scope of this study, these and other applications would benefit from practical guidance to minimize the likelihood and magnitude of exposure measurement error, and to understand how this affects the design and interpretation of studies. Rigorous and reproducible life course exposure estimates can only be developed with an understanding of several complex and nuanced issues. 

### 4.1. Residential Mobility of an Older Cohort

The present study appears to be the first to examine the mobility of a mid- to late-life cohort over long exposure windows. Residential history has been examined most frequently for pregnancy cohorts, where the number of residences or a missed residence during reproductive age can raise concerns for studies examining reproductive health and birth outcomes [21,46]. Our ALS cohort differed substantially from a pregnancy cohort: individuals were older; most have moved multiple times; exposure windows of interest were longer; issues regarding recall and data completeness were more pertinent, especially for earlier homes; and both males and females were included. For older adults, moving residences could be triggered by retirement, lifestyle factors, widowhood, health deterioration and disability, and social conditions [47,48]. National level statistics from the American Community Survey showed that 13% of Americans moved between 2017 and 2018, and that mobility declined with age (e.g., 25% of individuals aged 18–24 moved compared to 6% of individuals aged 65 and over) [49]. While we observed similar trends, suggesting our results were broadly representative, our largely White and relatively high socioeconomic status (SES) cohort was not necessarily representative of other racial/ethnic backgrounds and lower SES groups. The national survey also showed that mobility depended on rent/homeowner status (24% of renters moved annually compared to 6% of homeowner households) and income (14% in the bottom income quartile compared to 11% in the top quartile); factors not explored in the present study. ACS data for 2018–2019 also showed that 65% of moves were within the same county, 17% were between counties in the same state, 14% were between states, and 4% were from abroad. We did not investigate the distances of moves, but even short moves can affect exposure. In general, exposures among cohort members for different exposure windows were positively correlated, but the correlation decreased as exposure windows become further apart. Complete residential histories were needed to minimize exposure measurement errors.

### 4.2. Resolving Incomplete Residential Histories

Omissions and errors in residence time history and geocoding, a basic but important problem, will lead to reduced sample size and can result in selection bias, geographic bias, and reduced efficiency, particularly if the non-geocoded addresses are excluded [50,51]. Missing, incomplete, erroneous, and incompatible data mostly results from documentation or recall failures, data entry errors, and a lack of standardization in address formats [19]. Omissions and errors for street names, house numbers, ZIP codes, and place names lead to imprecise or incorrect mapping of locations, or a failure to geocode. While rigorous data cleaning, preprocessing, editing and verification of addresses can improve geocoding performance, 10 to 30% of addresses, and even more in specific subgroups, will be improperly geocoded [51]. Our dataset, even after extensive review and follow-up, had comparable rates. Omissions and errors tended to increase with longer time windows. 

We showed that a simple strategy could obtain virtually complete time histories from survey data. By adding a few additional questions, replacing missing data with available data from the preceding or following residence, making a few assumptions reflecting the most common months for moving, and utilizing a gap analysis to guide and check revisions, we obtained nearly 99% completion. The remaining gaps mostly occurred for residences occupied when participants were very young. 

For about 20% of the addresses (mostly the older ones), participants could not recall the move-in or move-out month. Since the duration between most moves was long (averaging over 8 years in our cohort), the precise start or end date is unlikely to be critical in most cases. Additionally, because many geospatial datasets provide only annual average data, including traffic data like AADT and the air pollution datasets used (38), within-year temporal trends were not captured. However, this issue could cause exposure measurement errors for short duration residences with an unknown date for exposure types and datasets that have significant seasonal variation, e.g., ozone (O_3_), which typically increases in summer and drops in winter. Errors might increase further if the exposure metric uses a maximum level, e.g., the 8-hour O_3_ National Ambient Air Quality Standard (NAAQS). In this case, the assumption of a mid-summer move (given a missing date) might overestimate exposure if the actual move occurred in the fall or winter. This situation might be addressed with datasets that provide the necessary temporal resolution using “temporal imputation,” e.g., randomly sampling over the possible time window (possibly the entire year). This imputation could also affect the exposure estimate for the preceding or following residence, possibly causing additional variation. 

We also demonstrated a spatial-multiple-interpolation approach designed to cope with missing or incomplete address data. This approach performed well and avoided issues associated with simpler centroid approaches. While somewhat intensive, it appears amenable to automation. We also noted the need to review match quality and to confirm results using several geocoding engines. These steps boosted geocoding success to 91.7% of the addresses provided by participants, compared to 67.4% based on survey responses alone. This high rate of success required manual review and interventions, and again, we note a potential role for machine learning/artificial intelligence.

We did not utilize or assess commercial services that provided residential address information using administrative databases. One assessment using a commercial service and a Michigan-based cohort matched 71.5% of the participants’ lifetime addresses obtained from a survey [52]. While not matching the completeness attained in our survey, these services have utility, particularly if survey data is unavailable. Additional concerns in using commercial services beyond completeness include the transparency needed to check or confirm results, the nature of data gaps (e.g., data missing not at random), uncertainty, and potentially prohibitively high costs. Additionally, providers like ArcGIS World Geocoding Services are designed to provide up-to-date address data but not historical address records, which may decrease the reliability of geocoding for older addresses.

### 4.3. Geospatial Datasets

Geospatial datasets can be diverse and heterogenous [53], differing by data type (e.g., point, nonpoint, linear, areal, volumetric features), format (e.g., point, line, polygon, raster), spatial and temporal resolution, spatial and temporal coverage, accuracy, and interpretation [5,54]. The five datasets examined here showed different spatial patterns; the pollutant measures (PM_2.5_, NO_2_ and BC) reflected differences in emission sources, dispersion, and atmospheric lifetimes of these pollutants, while TI measures were surrogates for on-road mobile source emissions. PM_2.5_ had the most complete monitoring dataset, and concentration gradients tended to be relatively smooth and reflected broad industrial, urban and rural differences. PM_2.5_ and NO_2_ levels in the geospatial datasets had reasonable agreement with monitoring data across the US, although a substantial fraction of sites were underpredicted and the variability was compressed. For BC, agreement was marginal to poor. These trends apply to annual averages – variability of daily and short-term levels will be much greater. Unsurprising, levels of the three pollutants and the two TI measures had moderate to high correlation. While the TI measures could account for spatial resolution finer than 1 km and targeted a particular source (on-road vehicles), specialized and yet non-routine modeling is needed to estimate air quality impacts from road network and vehicle volume data [55,56]. 

Air quality monitoring networks are too sparse spatially to represent spatial variability at the local scale for many pollutants and emission sources. This applies to traffic-related air pollutants, e.g., BC, ultrafine particles, and a fraction of NO_2_. Levels of these pollutants can increase considerably within a few hundred meters of major roadways, e.g., roadway increments ranged from 37% to 78% of the total BC level based on 2016 to 2018 US data, and PM_2.5_ increased by 1.3% to 27.1% [57,58,59]. We saw similar increases in BC near roads, with intermediate increases for NO_2_, but little effect for PM_2.5_. Such local variation occurred at a fraction of the range (spatial length) of the semivariograms derived with the geospatial datasets—these features cannot be represented at the 1 km resolution of these datasets. Consequently, the geospatial data was compressed and inaccurately portrayed roadway increments, a deficiency that limited its ability to represent exposures for individuals living near major roads or other large pollution sources, which are effects that can be important for some of the most exposed and disproportionately affected groups. Small-scale spatial heterogeneity can be even more pronounced for other types of exposures, such as strong and localized sources of air pollutants (e.g., sulfur dioxide (SO_2_), hydrogen sulfide (H_2_S), ethylene oxide (EtOH), and carbon monoxide (CO)), as well as noise, vibration, and heat. If the geospatial dataset were to account for this variation, then very accurate and precise geocoding would be needed. In many cases, however, the available geospatial datasets will have a limited ability to portray small-scale spatial variation and downscaling improvements will be marginal, potentially resulting in significant exposure measurement error for the most exposed groups. Still, the suggested approaches can improve completeness, reliability, and potentially the accuracy of the exposure assessment.

The geographic literature recognizes how smoothing or aggregation at larger length or area scales strengthens bivariate relationships [60]. This was reflected by higher correlation across pollutants in state-wide as compared to the urban scale analyses, and by the similar performance of the interpolation methods. The potential for exposure measurement error and bias due to insufficient spatial resolution in geospatial datasets is an important and underappreciated issue. The recent development of pollution datasets with 50 m resolution in urban areas [61,62] should improve accuracy for traffic-related pollutants. This problem applies to pollutants that can have strong and localized gradients due to nearby, strong, and often ground-level sources of emissions (e.g., CO, SO_2_, NO_2_, and EtOH just mentioned) as well as pollutants with very short residence times (e.g., ultrafine particulate matter, coarse-fraction-particulate matter, and dust). 

Geospatial exposure analyses have focused on chronic exposure, and thus have used long term averages, appropriate if the health response is driven by chronic or accumulated exposures. However, short-term information is needed in other applications, for example: the assessment of acute effects (e.g., asthma exacerbations driven by 1-hour maximum O_3_ or SO_2_ concentrations); exposure windows reflecting short periods of increased vulnerability; the analyses of episodic events (e.g., wildland fires and industrial disasters); and the analyses of specific periods and seasons (e.g., to account for outdoor daytime sports and exercise). The concentration metrics to describe these situations could be derived from time-resolved geospatial data, e.g., event maximum (a worst-case scenario), a short-term daily average, or an upper percentile level. While beyond the scope of the current analysis, their evaluation is likely more challenging than the chronic estimates examined here.

### 4.4. Spatial Linkage, Interpolations and Resolution 

A residence or other location can be spatially linked to exposure datasets using many techniques. While the nearest neighbor and spatial coincidence are commonly used, and at least partially account for the distance from the pollution source [27,28,63,64], these techniques can suffer from spatial bias, a lack of granularity, and artificial discontinuities. Such concerns pertain to all proximity, zonal, or buffer-based approaches using any specific region or distance around the location of interest [65]. While other studies have utilized 5 km buffers around residences (and 20-year histories), e.g., [66], the influence of buffer size warrants examination as it can critically affect results. In general, larger buffer size increases the number of individuals considered to be exposed and decreases exposure contrasts, while smaller buffers yield fewer exposed individuals and increase the significance of positional errors (see below). Interpolations using IDW, kriging, RBF, splines, natural neighbor, and Thiessen polygons may help avoid these problems, and can also utilize the spatial correlation often seen between nearby locations [67]. The selection and performance of interpolation schemes depend on the dataset characteristics, including its spatial representativeness, spatial dependencies, sampling density, and design [29,68,69,70,71]. Kriging has been a preferred interpolation approach for air pollutants [29,30], outperforming the nearest neighbor approach for PM_2.5_ [72] and IDW approaches for PM_2.5_ and NO_2_ [73,74]. We saw little difference among the interpolation techniques with the geospatial datasets, but this resulted from spatial averaging across the pixel area (1 km^2^) that did not permit finer-scale features to be resolved, like near-road increments.

The spatial resolution of the geospatial dataset effectively sets accuracy and precision criteria for geocoding. For the air pollution datasets in the present study, obtaining geocoordinates within 1 km of the true location was sufficient, and higher resolution would confer little additional benefit. In urban areas, this size roughly corresponds to the size of a block group. (The areas of the census divisions are highly variable and much larger in rural areas.) Census tracts and 5-digit ZIP codes, even in urban areas, do not have the desired precision. The routing information on a 9-digit ZIP code (resolvable to a ZIP code tabulation area) would typically meet the 1 km criterion. Anticipating the emergence of higher resolution datasets; however, and considering metrics like TI and effects like roadway increments, geocoding to 10 to 50 m accuracy is recommended, a precision comparable to the positional error that can obtained using automated geocoders [75].

### 4.5. Limitations and Evaluation of Geospatial Exposure Estimates 

Geospatial exposure data have had many successful applications [5], but their limitations should be recognized. First, geospatial data provide proxy or indirect measures of external exposure. An individual’s actual exposure, i.e., uptake or dose, depends on the exposure route (inhalation, ingestion, or dermal), dosimetry parameters (e.g., breathing rate), time–activity data (locations and time spent in each), and effects of indoor, occupational, vehicular, and other compartments that can affect concentrations and uptake [3,76]—all of which are factors not reflected in geospatial data. Thus, geospatial exposure estimates will not necessarily correspond to field data, a traditional definition of model validation [77]. Comparison of geospatial exposure estimates generated by different studies or by different methods essentially represent model-to-model comparisons, a step in the broader model evaluation process and model “lifecycle” [78]. To be comprehensive and realistic, evaluations should include the consideration of the issues raised in the present analysis, including the completeness and accuracy of the residential history, and the spatial resolution of the exposure dataset. In this study, we proposed and separately evaluated several approaches to improving the completeness, reliability, and accuracy of geospatial data for the life course exposure estimates and used an approach that illustrated the effect of each step. Comparisons with other datasets, and potentially with key outcome measures, would help ensure our results are generalizable.

A second limitation for geospatial exposure estimates is spatial resolution. As noted above, a resolution of 1 km is insufficient to represent small-scale spatial heterogeneity, e.g., roadway increments and other localized effects, resulting in systematic underprediction for the most exposed individuals and groups. A third limitation is that many geospatial datasets do not portray temporal variation, which can occur at hourly, diurnal, and seasonal levels [79,80]. While long-term or average information is often desired, temporal information is needed to incorporate mobility and time–activity data that could refine exposures, e.g., accounting for outdoor exercise and commuting. Temporal information is also needed for longitudinal applications (e.g., brief but intense exposure to wildland fire smoke and acute health outcomes), more gradual but meaningful changes in traffic-related pollutants (e.g., from vehicle volume growth, fleet mix changes, and new emission controls), and the consideration of exposure sequences (e.g., whether a PM_2.5_ episode preceded or followed an infectious disease outbreak). Fourth, while not confined to geospatial data, specific pollutants like PM_2.5_ should be recognized as indicators of mixtures that can vary temporally and spatially in composition, size, and other characteristics [81,82]. While not a limitation of geospatial data itself, a fifth factor concerns the dependence of exposure and other risk and mediating factors on age and sex, thus epidemiological applications of life course exposure estimates will likely need age- and sex-matched controls, appropriate stratification, or other adjustments.

These issues above, plus the spatial resolution and period available, can affect the selection of exposure datasets appropriate for life course exposure studies. We anticipate that both the number and quality of candidate datasets will continue to increase. While dataset refinements may not alter the fundamental limitations as an exposure proxy, improvements will improve the ability to characterize external exposures, an exciting development given the many built, natural, and social features that have been mapped to assess the role of environmental and community exposure on health [4]. 

## 5. Conclusions

Geospatial datasets have become essential and widely used in risk, epidemiology, and other applications, enabling new discoveries that increase our understanding of environmental risk factors and potentially our ability to manage exposures to improve public health. This paper has addressed several key issues encountered in using these data to develop life course exposure estimates with the goal of providing practical guidance and solutions for researchers to improve exposure assessments. The suggested steps to improve the completeness of residential time and location histories, confirm and impute missing date and location data, and better link exposure datasets to residential locations are broadly applicable and can improve the completeness, reliability, and accuracy of exposure assessments. The analysis also highlights the need for datasets with high spatial resolution to represent exposure types with small-scale spatial heterogeneity, e.g., near-road increments of traffic-related air pollutants like black carbon, and datasets that incorporate temporal resolution sufficient to represent daily, seasonal, and decadal variation, e.g., important for noise and pollutants like O_3_ and PM_2.5_. These steps will improve life course exposure estimates and ultimately lead to actions that reduce exposure, risk, and disease.

## Figures and Tables

**Table 1:** Hierarchy of data to obtain an initial estimate of move-in and move-out dates for each residence. N is number of cases where hierarchy steps applied in test dataset.

Seq.	Year Move-In Hierarchy	N	(%)	Seq.	Year Move-Out Hierarchy	N	(%)
1	Move-in year	8731	(88.5)	1	Move-out year	7565	(76.7)
2	Age at move-in + year of birth	806	(8.2)	2	Age at move-out + year of birth	845	(8.6)
3	Prior move-out year	16	(0.2)	3	Following move-in year	68	(0.7)
4	Prior move-out age + year of birth	3	(0.0)	4	Following move-in age + year of birth	5	(0.1)
	For first (childhood) residence, birth year	171	(1.7)	5	For last residence, year of death	466	(4.7)
5	Missing	134	(1.4)	6	For last residence, current year (living)	779	(7.9)
	Total	9861	(100.0)	7	Missing	133	(1.3)
					Total	9861	(100.0)
Seq.	Month Move-In Hierarchy	N	(%)	Seq.	Month Move-Out Hierarchy	N	(%)
1	Move-in month	7572	(76.8)	1	Move-out month	6472	(65.6)
2	Prior move-out month	68	(0.7)	2	Following move-in month	223	(2.3)
3	Initial default (8 = Aug)	2221	(22.5)	3	For latest residence, month of death	475	(4.8)
	Total	9861	(100.0)	4	For latest residence, current month (living)	780	(7.9)
				5	Initial default (7 = July)	1911	(19.4)
					Total	9861	(100.0)

**Table 2:** Summary of average number of residences per person for lifetime and for 20-year exposure window, grouped by relationship status. N = number of individuals.

Relationship Status	Males			Females			Total		
	Life	20 Years	N	Life	20 Years	N	Life	20 Years	N
Married, Partner	7.50	2.00	575	7.69	1.98	390	7.58	1.99	965
Divorced, Separated	7.00	2.71	76	7.52	2.71	95	7.29	2.71	171
Widowed	7.44	1.56	16	7.09	2.18	57	7.16	2.04	73
Never married	7.66	3.07	44	8.20	2.93	44	7.93	3.00	88
NA	8.17	3.00	6	6.75	1.50	4	8.40	2.40	10
All	7.46	2.14	717	7.64	2.18	590	7.55	2.16	1307

**Table 3:** Summary of geocoding and imputation used to complete residence history. NA is not applicable.

Information Category		Coordinate Extraction	Length (km) or Area (km^2^)	Residence Location		Total
				Michigan	Elsewhere US	
1: Sufficient Information		Geocoded	NA	2391	557	2948
2: Partial Information						
	2A: Info. Corrected	Geocoded	NA	40	9	49
	2B: Info. Corrected (Small Street/Area)	Approximated	Length/Area< 1	17	8	25
	2C: Small Street/Area	Approximated	Length/Area< 1	258	100	358
	2D: Addresses with Street Information		1≤ Length< 2	79	42	121
		Multiple	2≤ Length< 5	122	35	157
		Imputation	5≤ Length< 10	53	14	67
			10≤ Length≤ 20	53	7	60
			Length >20	0	2	2
	2E: Addresses with Area Information		1≤ Area< 2	7	11	18
		Multiple	2≤ Area< 5	22	26	48
		Imputation	5≤ Area< 10	27	22	49
			10≤ Area≤ 20	43	45	88
			Area >20	11	11	22
3: Inadequate Information		NA	NA	148	217	365
Total		NA	NA	3271	1106	4377

**Table 4:** Summary of test datasets and comparison of actual and imputation results. M-W is the Mann–Whitney U *p*-value. K-S is the Kolmogorov–Smirnov *p*-value. N = 50.

Exposure Metric	Type	Summary Statistics				Spearman	Distribution Tests	
		Mean	St. Dev.	Minimum	Max	R	M-W	K-S
PM_2.5_ (μg/m^3^)	Actual	7.49	1.63	4.72	10.01	0.98	0.99	1.00
	Imputed	7.48	1.64	4.74	10.10			
BC (μg/m^3^)	Actual	0.58	0.14	0.36	0.85	0.98	1.00	1.00
	Imputed	0.58	0.14	0.35	0.85			
NO_2_ (ppb)	Actual	5.99	3.31	1.61	13.54	0.99	0.88	1.00
	Imputed	5.94	3.33	1.60	13.28			
LDV TI	Actual	126,376	144,107	221	529,330	0.99	0.97	1.00
	Imputed	128,497	146,910	130	542,061			
HDV TI	Actual	9956	10,242	11	33,915	0.98	1.00	1.00
	Imputed	9982	10,030	7	32,799			

**Table 5:** Spearman correlation coefficients for exposure metrics at 1 km grid level for Michigan and 2016. Colors show heat map according to scale at right.

Michigan (N = 141,065)						Southeast Michigan (N = 24,630)						
	PM_2.5_ (μg/m^3^)	BC (μg/m^3^)	NO_2_ (ppb)	LDV TI	HDV TI		PM_2.5_ (μg/m^3^)	BC (μg/m^3^)	NO_2_ (ppb)	LDV TI	HDV TI	Color Scale
PM_2.5_ (μg/m^3^)	1.00					PM_2.5_ (μg/m^3^)	1.00					0.00
BC (μg/m^3^)	0.93	1.00				BC (μg/m^3^)	0.80	1.00				0.25
NO_2_ (ppb)	0.70	0.66	1.00			NO_2_ (ppb)	0.58	0.46	1.00			0.50
LDV TI	0.67	0.61	0.72	1.00		LDV TI	0.39	0.23	0.78	1.00		0.75
HDV TI	0.64	0.58	0.71	0.96	1.00	HDV TI	0.40	0.25	0.77	0.92	1.00	1.00

**Table 6:** Semivariogram characteristics for five exposure metrics and spherical, exponential, and Gaussian correlation functions. Developed using 1 km resolution geospatial data for 2016, 100 lags, and lag distance of 1500 m. Model fit is based on examination of semiovariograms. Highlights show best fit case.

Exposure	Parameter		Southeast Michigan			Michigan	
Metric		Spherical	Exponential	Gaussian	Spherical	Exponential	Gaussian
PM_2.5_	Range (km)	59	78	35	150	150	39
	Partial Sill	1.56	1.64	1.50	0.49	0.45	0.37
	Model Fit	Good	Fair	Fair	Good	Fair	Poor
	Symmetry	Asymmetric	Asymmetric	Asymmetric	Asymmetric	Asymmetric	Asymmetric
	RMSE	0.16	0.16	0.25	0.07	0.07	0.08
BC	Range (km)	60	81	34	71	131	19
	Partial Sill	0.013	0.014	0.012	0.003	0.004	0.002
	Model Fit	Good	Poor	Poor	Fair	Good	Very Poor
	Symmetry	Asymmetric	Asymmetric	Asymmetric	Asymmetric	Asymmetric	Asymmetric
	RMSE	0.023	0.023	0.024	0.015	0.015	0.016
NO_2_	Range (km)	150	150	43	77	140	40
	Partial Sill	22	15	10	11	13	10
	Model Fit	Good	Fair	Poor	Good	Fair	Fair
	Symmetry	Asymmetric	Asymmetric	Asymmetric	Asymmetric	Asymmetric	Asymmetric
	RMSE	0.42	0.42	1.94	0.43	0.43	1.92
LDV	Range (km)	64	93	45	78	116	46
TI	Sill (×10^6^)	17,391	18,839	17,190	1912	2058	1843
	Model Fit	Fair	Fair	Good	Very Good	Good	Fair
	Symmetry	Asymmetric	Asymmetric	Asymmetric	Symmetric	Symmetric	Symmetric
	RMSE	10,465	10,467	15	3432	3432	2562
HDV	Range (km)	62	84	32	55	85	27
TI	Sill (×10^6^)	65	68	58	8	9	8
	Model Fit	Good	Good	Fair	Good	Very Good	Fair
	Symmetry	Symmetric	Symmetric	Symmetric	Symmetric	Symmetric	Symmetric
	RMSE	872	872	1346	330	330	400

## Data Availability

The authors endeavor to share, to the extent possible, data and code that is not protected. Please contact the corresponding author.

## Supplementary Materials

Supporting information referred to in the text can be downloaded at: https://www.mdpi.com/article/10.3390/ijerph22111629/s1. Figure S1: Maps showing locations of participants by county in continental US and in Michigan, Figure S2: Two visualizations of residence location time history for the same study participant, Figure S3: Number of residences reported by decile of participant age and sex, Figure S4: Distribution of actual and imputed exposures for PM2.5, BC and NO2, LDV TI and HDV TI. N = 50 validation locations, Figure S5: Scatterplots of geospatial estimate of BC versus monitored data for years 2000–2003 and for individual years from 2004–2015, Figure S6: Scatterplots of geospatial estimate of BC versus monitored data for years 2000–2003 and for individual years from 2004–2015, Figure S7: Scatterplots showing monitored concentrations of BC versus distance from the closest major highway, Figure S8: Bland-Altman plots evaluating agreement between geostatistical estimates and monitoring observations of PM2.5, BC and NO2, Figure S9: Distributions of coefficient of variance (COV) for geospatial data within 2.5 km of EPA monitoring sites (16 pixels) for PM2.5, BC and NO2, Figure S10: Scatterplots showing coefficient of variation for geospatial data within 2.5 km of EPA monitoring sites (16 pixels) for PM2.5, BC and NO2, Table S1: Summary of participants in study, grouped by cases, controls, and at-risk, Table S2: Distance of EPA monitoring sites from nearby highway (Year = 2016), Table S3: Summary of concentrations and COVs for the monitoring site data, the 16 points of geospatial data closest to monitoring sites (within 2.5 km radius), and the 4 points of geospatial data within 1 km radius), Table S4: Performance of interpolation schemes for matching monitored data.

## Abbreviations

The following abbreviations are used in this manuscript:

AADT	annual average daily traffic
ALS	amyotrophic lateral sclerosis
BC	black carbon
CAADT	commercial annual average daily traffic
COV	coefficient of variation
CVKT	commercial vehicle kilometers traveled
HDV	heavy duty vehicle, e.g., truck
IDW	inverse distance weighting
KS	Kolmogorov–Smirnov
LDV	light duty vehicle, e.g., car
LUR	land use regression
MAE	mean average error
MAPE	mean absolute percentage error
MI	multiple imputation
NAAQS	National Ambient Air Quality Standard
NO2	nitrogen dioxide
PM2.5	particulate matter under 2.5 micron in diameter
RBF	radial basis function
RMSE	root mean square error
SES	socioeconomic status
TI	traffic intensity
VKT	vehicle kilometers traveled
