# Supplementary material for: Development of Life Course Exposure Estimates Using Geospatial Data and Residence History
Source: Int J Environ Res Public Health. 2025 Oct 26;22(11):1629. doi: 10.3390/ijerph22111629 (PMC12653017; doi:10.3390/ijerph22111629)
Supplement: Supplementary file 1 [file ijerph-22-01629-s001.zip › ijerph-3881752-supplementary.pdf]

Development of Retrospective Exposure Estimates Using Geospatial Data and Residence History

Stuart Batterman, Md Kamrul Islam, Stephen Goutman

Supplemental Information

October 8, 2025

CONTENTS

Figure S1. Maps showing locations of participants by county in continental US and in Michigan

Figure S2. Two visualizations of residence location time history for the same study participant

Figure S3. Number of residences reported by decile of participant age and sex.

Figure S4. Distribution of actual and imputed exposures for $PM_{2.5}$, BC and NO_2, LDV TI and HDV TI. N=50 validation locations.

Figure S5. Scatterplots of geospatial estimate of BC versus monitored data for years 2000-2003 and for individual years from 2004-2015

Figure S6. Scatterplots of geospatial estimate of BC versus monitored data for years 2000-2003 and for individual years from 2004-2015

Figure S7. Scatterplots showing monitored concentrations of BC versus distance from the closest major highway

Figure S8. Bland-Altman plots evaluating agreement between geostatistical estimates and monitoring observations of $PM_{2.5}$, BC and NO_2.

Figure S9. Distributions of coefficient of variance (COV) for geospatial data within 2.5 km of EPA monitoring sites (16 pixels) for $PM_{2.5}$, BC and NO_2

Figure S10. Scatterplots showing coefficient of variation for geospatial data within 2.5 km of EPA monitoring sites (16 pixels) for $PM_{2.5}$, BC and NO_2

Table S1. Summary of participants in study, grouped by cases, controls, and at-risk.

Table S2. Distance of EPA monitoring sites from nearby highway (Year =2016)

Table S3. Summary of concentrations and COVs for the monitoring site data, the 16 points of geospatial data closest to monitoring sites (within 2.5 km radius), and the 4 points of geospatial data within 1 km radius)

Table S4. Performance of interpolation schemes for matching monitored data

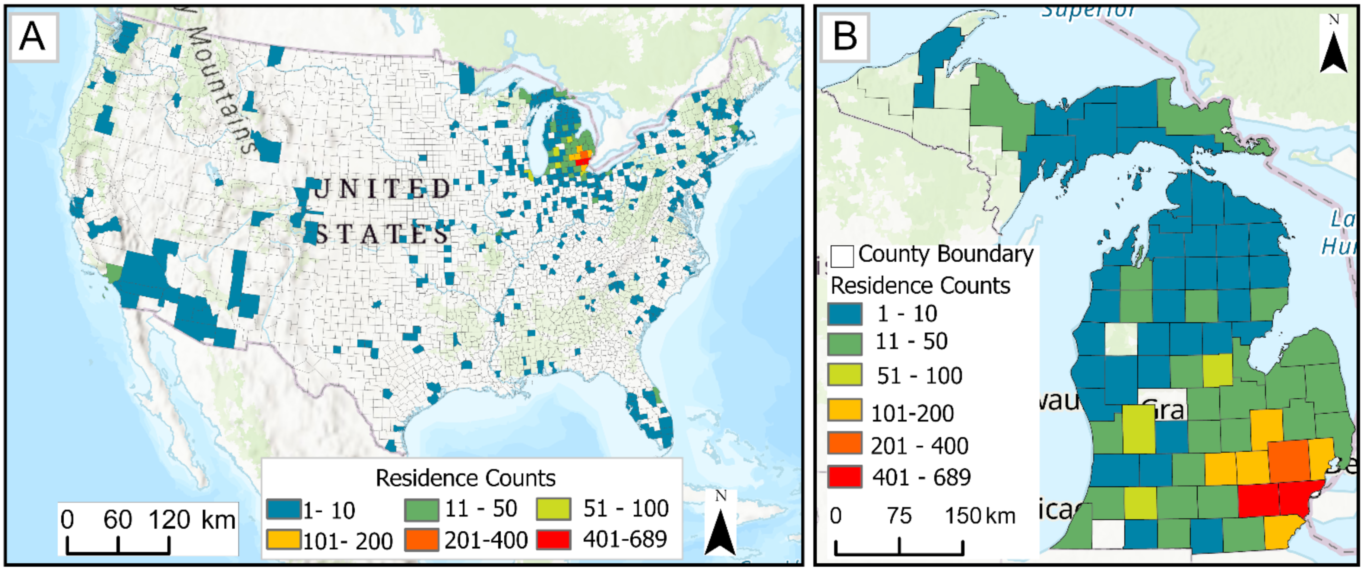

Figure S1. Maps showing locations of participants by county in continental US (left) and in Michigan (right). Total of 4012 locations in US and 889 in Michigan.

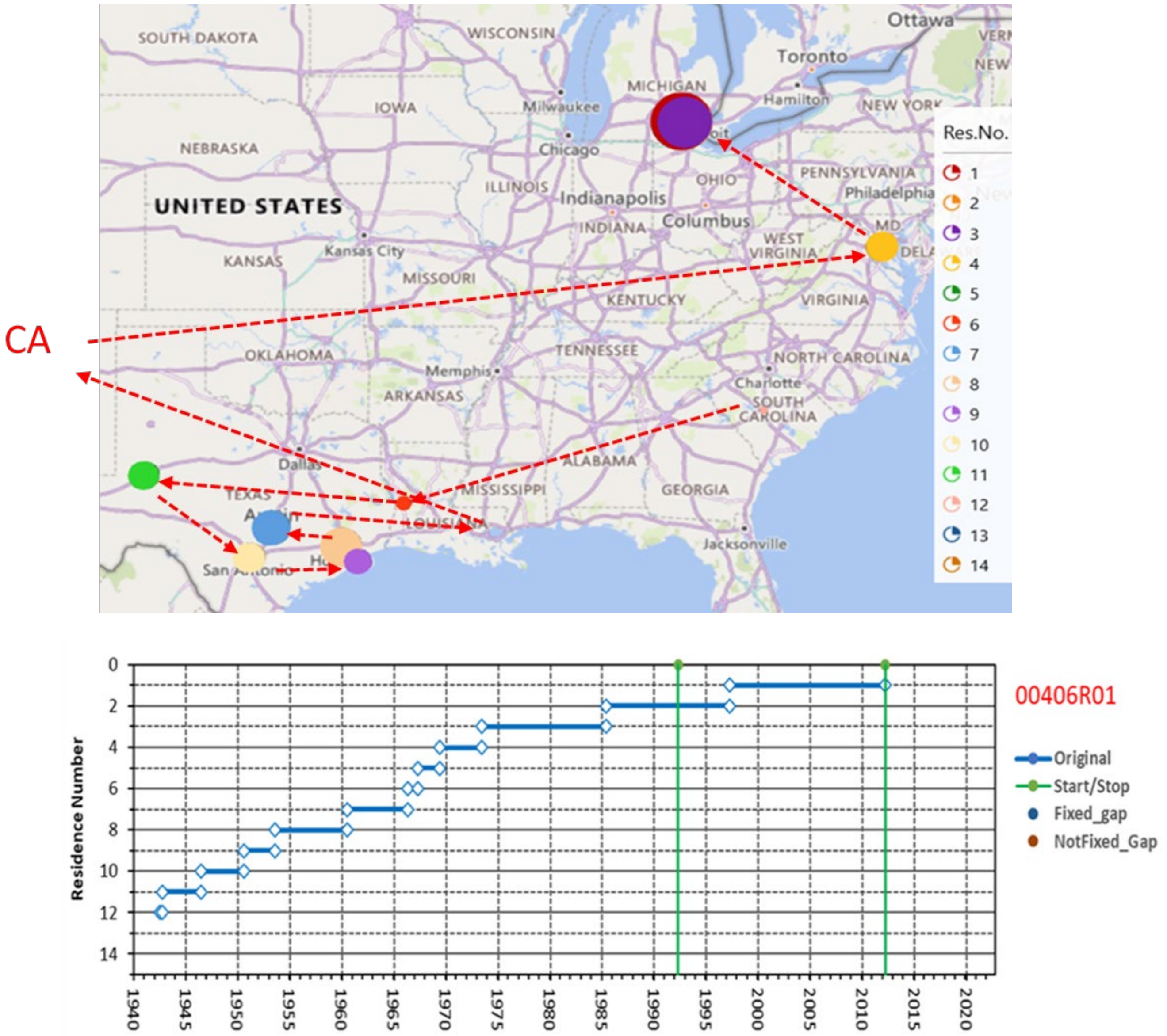

Figure S2. Two visualizations of residence location time history for the same study participant who reported 12 prior addresses over 70 years. Top: trajectory of moves on map showing birth place in South Carolina, then moving to Texas (5 locations), then to Louisiana, then California, then Virginia, then Michigan (3 locations). Bottom: Move-in and move-out dates for each residence for the participant. The longest residence period was 15 yearsl the shortest was 0.25 year. This participant had a complete record. Green lines indicate 20 year period prior to end of record. Gaps and overlaps are easilty seen in this presentation.

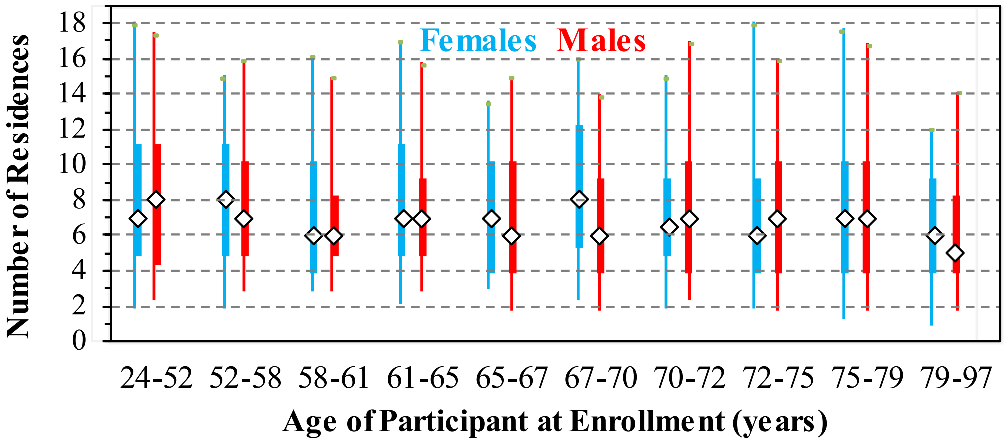

Figure S3. Number of residences reported by decile of participant age and sex. Plots shows 5th, 25th, 50th, 75th and 95th values. N=131 in each decile (combined male and female).

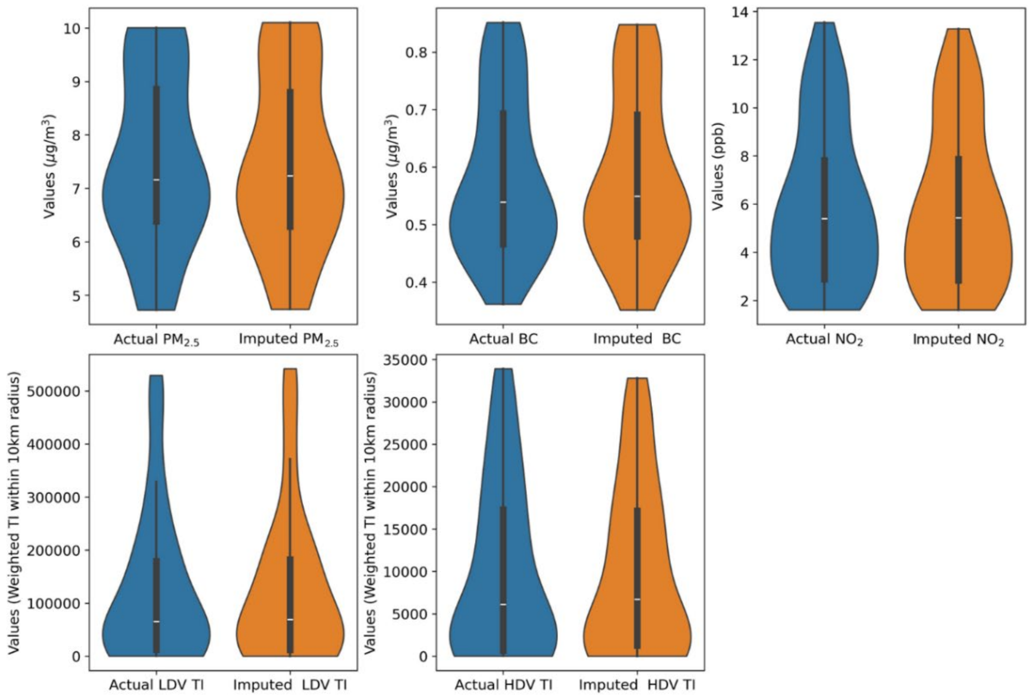

Figure S4. Distribution of actual and imputed exposures for $PM_{2.5}$, BC, NO_2, LDV TI and HDV TI. N=50 validation locations.

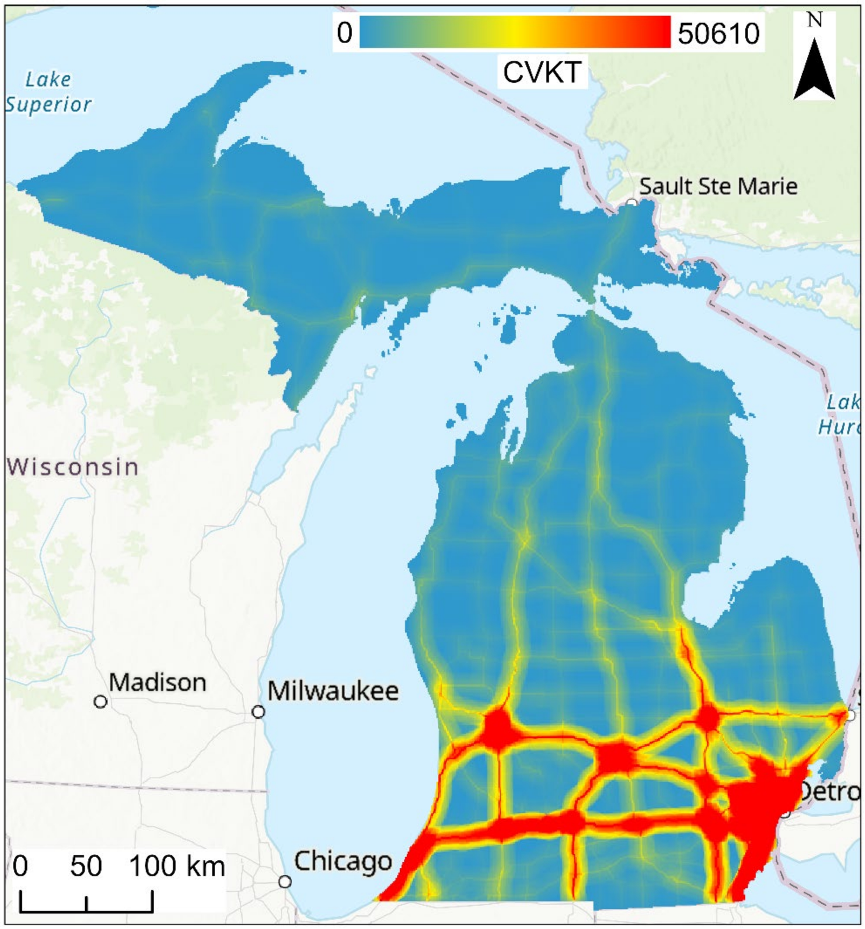

Figure S5. Maps showing levels of non-commercial traffic intensity (TI) across Michigan for 2016. TI uses inverse distance weighted VKT in a 10 km buffer.

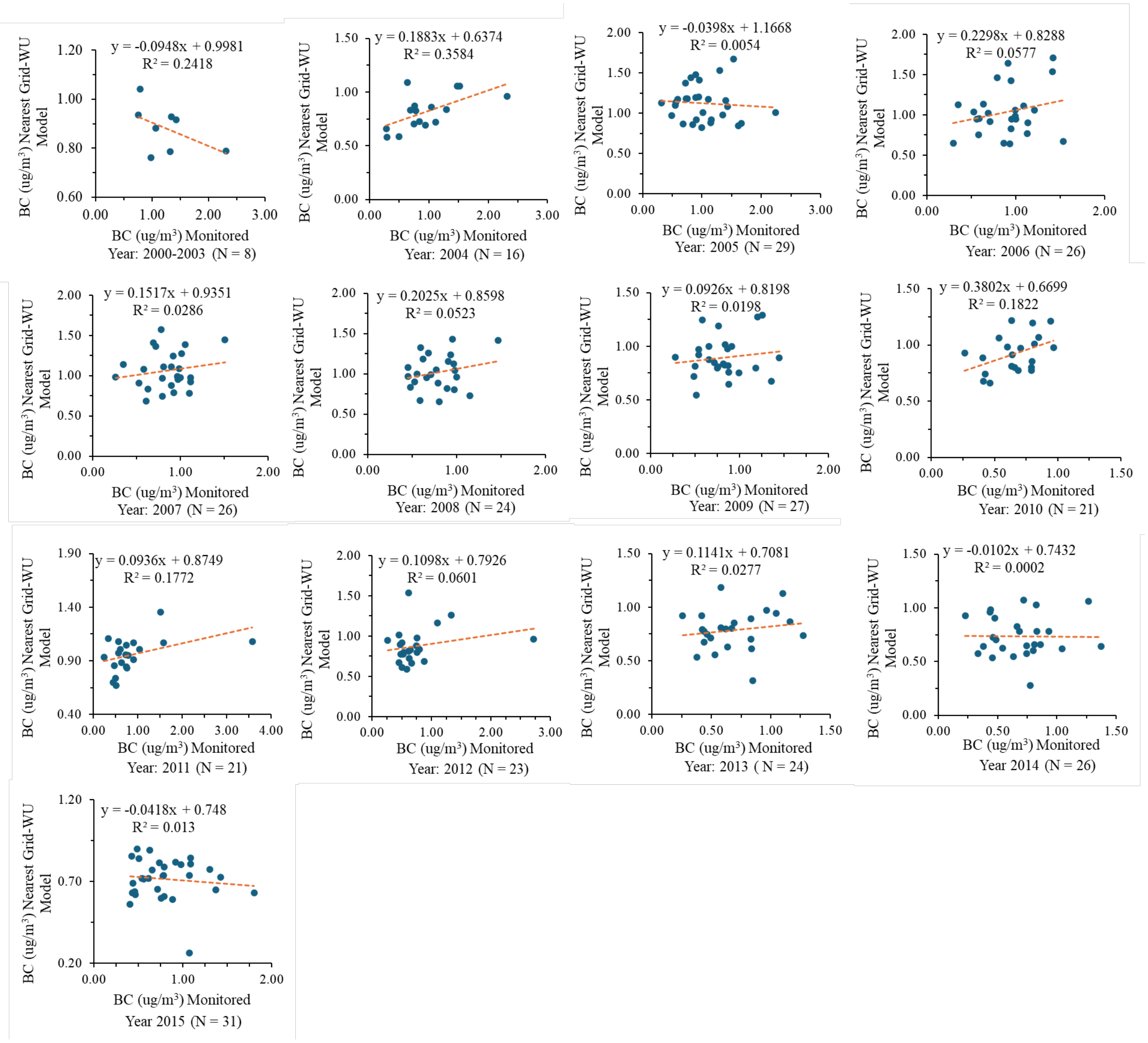

Figure S6. Scatterplots of geospatial estimate of BC versus monitored data for years 2000-2003 and for individual years from 2004-2015. Geospatial estimate from Washington University uses closest cell. Each plot shows regression line, R^2 and sample size N (number of sites). Sample size in years 2000, 2001, 2002 and 2003 is 1, 1, 2 and 4, respectively.

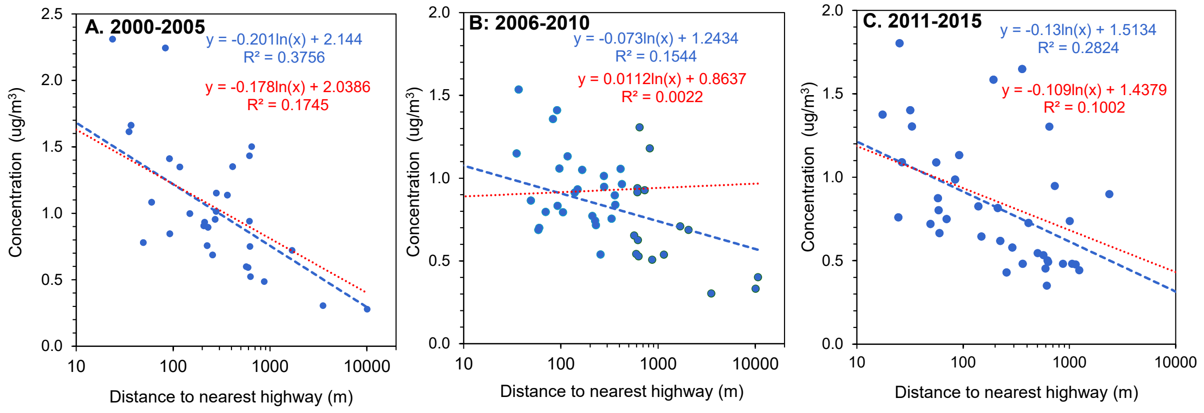

Figure S7. Scatterplots showing monitored concentrations of BC versus distance from the closest major highway. Plots show 5-year average (as available) for 2000-2005, 2006-2010, 2011-2015 periods. N is the number of sites.

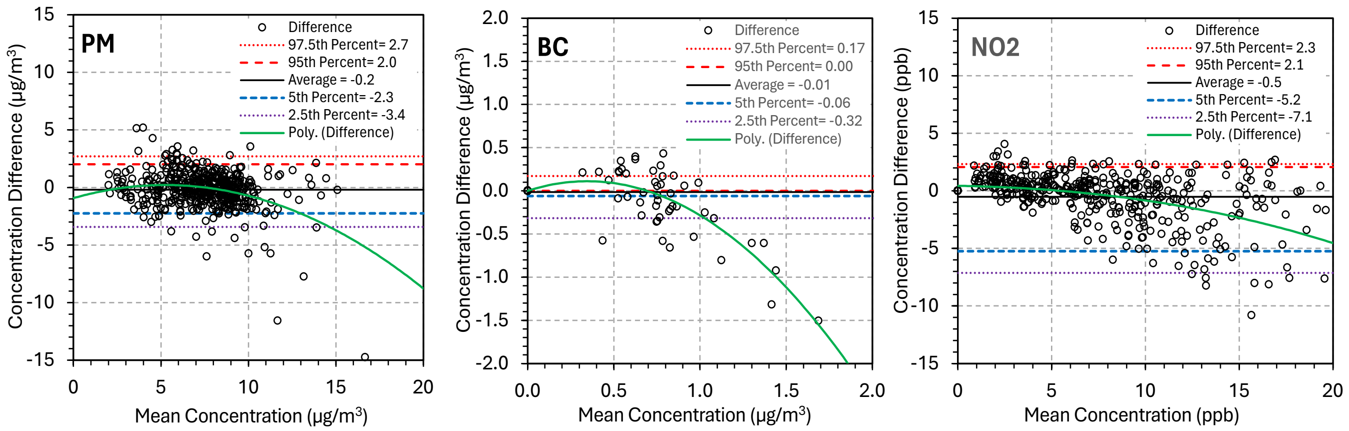

Figure S8. Bland-Altman plots evaluating agreement between geostatistical estimates and monitoring observations of $PM_{2.5}$, BC and NO_2.

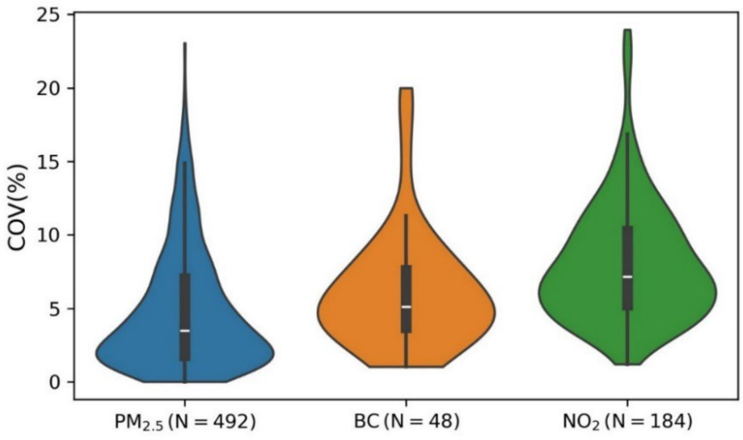

Figure S9. Distributions of coefficient of variance (COV) for geospatial data within 2.5 km of EPA monitoring sites (16 pixels) for $PM_{2.5}$, BC and NO_2. N is the number of monitoring sites.

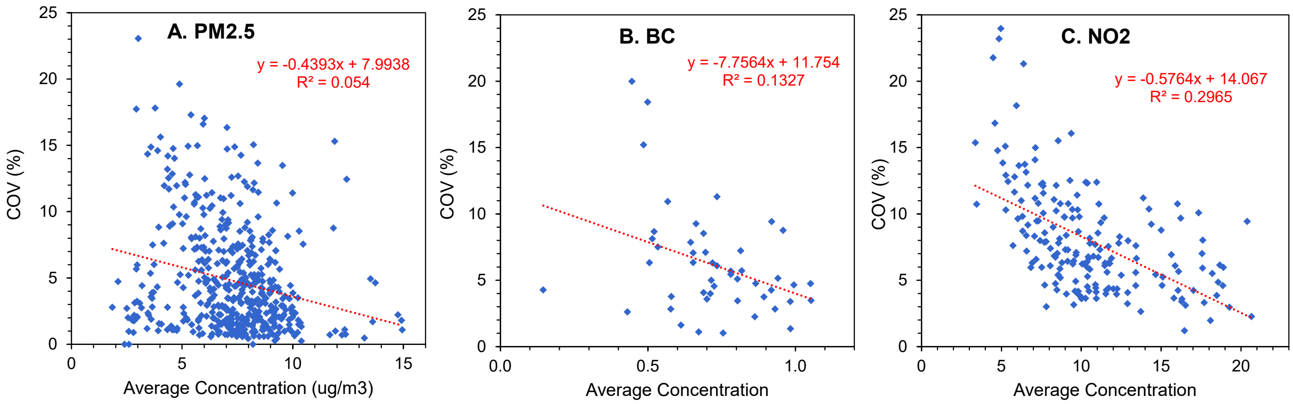

Figure S10. Scatterplots showing coefficient of variation for geospatial data within 2.5 km of EPA monitoring sites (16 pixels) for $PM_{2.5}$, BC and NO_2.

Table S1. Summary of participants in study, grouped by cases, controls, and at-risk. Also shows numbers married or with partner, divorced or separated, widowed and never married.

		Cases			Controls			At Risk			Average/Total		
		Male	Female	Both	Male	Female	Both	Male	Female	Both	Male	Female	Both
Age (years)	Average	63.9	65.7	64.7	61.5	59.5	60.6	64.6	61.4	63.0	63.0	62.6	62.8
	St.Dev.	11.6	9.8	10.9	10.8	12.6	11.8	10.2	9.7	10.1	11.3	11.4	11.3
	Minimum	22.3	39.6	22.3	22.5	20.3	20.3	34.1	24.7	24.7	22.3	20.3	20.3
	Maximum	92.7	90.2	92.7	89.5	82.7	89.5	82.3	81.5	82.3	92.7	90.2	92.7
Number	Married/Partner	320	196	516	200	150	350	55	44	99	575	390	965
	Divorced/Separated	32	31	63	40	53	93	4	11	15	76	95	171
	Widowed	7	37	44	8	17	25	1	3	4	16	57	73
	Never married	13	10	23	30	31	61	1	3	4	44	44	88
	NA	4	3	7	2	1	3	0	0	0	6	4	10
	Total	376	277	653	280	252	532	61	61	122	717	590	1307

Table S2. Distance of EPA monitoring sites from nearby highway (Year =2016)

	Monitoring Sites (%)		
Distance from Road (m)	$PM_{2.5}$ (N = 492)	BC (N = 48)	NO_2 (N = 409)
Distance ≤50	10.98	29.17	11.98
50 <Distance ≤ 100	10.37	22.92	15.16
100 <Distance ≤ 500	37.40	20.83	34.72
500 <Distance ≤ 1000	20.93	14.58	16.63
Distance >1000	20.33	12.50	21.52

Table S3. Summary of concentrations and COVs for the monitoring site data, the 16 points of geospatial data closest to monitoring sites (within 2.5 km radius), and the 4 points of geospatial data within 1 km radius). Concentrations for $PM_{2.5}$ and BC are ug/m^3 and in ppb for NO_2.

Pollutant	Statistic	Monitoring	16 points - 2.5 km			4 points - 1 km		
		Sites	Average	St.Dev.	COV	Average	St.Dev.	COV
$PM_{2.5}$	Average	7.6	7.3	0.3	**4.8**	7.4	0.2	**3.0**
	St.Dev.	2.5	2.1	0.3	4.0	2.1	0.2	3.1
	COV-Sites (%)	**33.7**	-	-	**29.3**	-	-	**28.9**
	Minimum	1.0	1.9	0.0	0.0	1.8	0.0	0.0
	25th Percentile	6.0	6.0	0.1	1.8	6.2	0.1	0.8
	50th Percentile	7.7	7.5	0.3	3.5	7.5	0.1	1.8
	75th Percentile	9.0	8.6	0.5	7.0	8.7	0.3	4.2
	90th Percentile	10.2	9.5	0.7	11.0	9.6	0.5	7.3
	99th Percentile	14.6	13.5	1.1	17.1	13.7	1.0	13.6
	Maximum	24.0	14.9	1.8	23.1	15.0	1.5	18.2
	N	492	492	492	492	492	492	492
BC	Average	0.9	0.7	0.0	**6.1**	0.7	0.0	**4.3**
	St.Dev.	0.5	0.2	0.0	4.0	0.2	0.0	3.6
	COV-Sites (%)	**52.6**	-	-	**25.7**	-	-	**25.3**
	Minimum	0.2	0.1	0.0	1.0	0.1	0.0	0.5
	25th Percentile	0.6	0.6	0.0	3.7	0.6	0.0	1.6
	50th Percentile	0.8	0.7	0.0	5.1	0.7	0.0	2.9
	75th Percentile	0.9	0.9	0.0	7.6	0.8	0.0	6.6
	90th Percentile	1.5	1.0	0.1	9.9	1.0	0.1	8.7
	99th Percentile	2.3	1.1	0.1	19.3	1.1	0.1	13.7
	Maximum	2.4	1.1	0.1	20.0	1.1	0.1	14.0
	N	48	48	48	48	48	48	48
NO_2	Average	8.3	10.5	0.8	**8.0**	10.5	0.6	**6.2**
	St.Dev.	5.8	3.8	0.3	**4.0**	3.8	0.4	5.1
	COV-Sites (%)	**69.2**	-	-	**36.1**	-	-	**36.0**
	Minimum	-0.2	3.4	0.2	1.2	3.7	0.1	0.4
	25th Percentile	3.9	7.7	0.5	5.2	7.8	0.3	3.3
	50th Percentile	7.5	9.8	0.7	7.1	9.9	0.5	4.7
	75th Percentile	11.4	12.2	0.9	10.2	12.6	0.7	8.0
	90th Percentile	16.1	16.3	1.2	12.7	16.4	1.1	11.3
	99th Percentile	25.7	19.5	1.7	22.0	19.7	1.5	29.4
	Maximum	30.9	20.7	1.9	24.0	20.6	2.0	38.6
	N	409	184	184	184	184	184	184

Table S4. Performance of interpolation schemes for matching monitored data. Includes root mean square error (RMSE), mean average error (MAE), mean absolute percentage error (MAPE) and R^2. Best metric for each pollutant is bolded. Number of monitoring sites: $PM_{2.5}$ = 492, BC = 48 and NO_2 = 409. Best parameter for each power/function is shown.

Pollutant	Interpolation Method	Power/Function	No. Points	RMSE	MAE	MAPE (%)	R^2
$PM_{2.5}$ (N = 492)	Nearest Point		1	1.659	1.095	17.437	0.584
	IDW	1	4	1.664	1.095	17.449	0.582
		1.5	4	1.664	1.095	17.471	0.582
		1.8	4	1.664	1.096	17.482	0.582
	Ordinary Kriging	Exponential	10	1.665	1.098	17.527	0.581
	Universal Kriging	Exponential; Gaussian	12	1.668	1.097	17.450	0.581
	RBF	Completely Regularized Spline	8	1.666	1.098	17.503	0.581
		Inverse Multiquadric	8	1.681	1.103	17.368	0.577
		Multiquadric	10	1.665	1.098	17.527	0.581
BC (N = 48)	Nearest Point		1	0.448	0.329	38.616	0.148
	IDW	1	8	0.447	0.327	38.446	0.150
		1.5	8	0.447	0.328	38.523	0.148
		1.8	8	0.447	0.328	38.539	0.148
	Ordinary Kriging	Gaussian	16	0.447	0.327	38.521	0.149
	Universal Kriging	Exponential; Gaussian	8	0.447	0.327	38.269	0.154
	RBF	Completely Regularized Spline	12	0.447	0.328	38.617	0.149
		Inverse Multiquadric	8	0.447	0.327	38.269	0.154
		Multiquadric	10	0.446	0.328	38.614	0.150
NO_2 (N = 409)	Nearest Point		1	2.530	1.657	38.049	0.837
	IDW	1	4	2.554	1.688	41.662	0.837
		1.5	4	2.542	1.676	41.194	0.839
		1.8	4	2.537	1.671	40.985	0.839
	Ordinary Kriging	Exponential	14	2.517	1.637	38.479	0.843
	Universal Kriging	Exponential; Exponential	16	2.686	1.756	44.668	0.822
	RBF	Completely Regularized Spline	8	2.622	1.749	46.481	0.828
		Inverse Multiquadric	8	2.695	1.838	52.204	0.817
		Multiquadric	14	2.505	1.621	36.243	0.845
